# Protein Corona Engineering of Lipid Nanoparticles Enables Efficient Functional Gene Silencing in the Liver

**DOI:** 10.1002/smll.75537

**Published:** 2026-09-15

**Authors:** Naths G. Sukubo, Serena Renzi, Martina B. Violatto, Leila Altigieri, Anna L. Capriotti, Andrea Cerrato, Aldo Laganà, Luca Digiacomo, Erica Quagliarini, Antonio Minopoli, Massimiliano Papi, Daniela Pozzi, Carlo A. Scirè, Pietro Invernizzi, Paolo Bigini, Giulio Caracciolo

**Affiliations:** ^1^ School of Medicine and Surgery University of Milano‐Bicocca Monza Italy; ^2^ Department of Molecular Medicine Sapienza University of Rome Rome Italy; ^3^ Department of Biochemistry and Molecular Pharmacology Istituto di Ricerche Farmacologiche “Mario Negri” IRCCS Milano Italy; ^4^ Department of Chemistry Sapienza University of Rome Rome Italy; ^5^ Interuniversity Consortium INBB – Biostructures and Biosystems National Institute Rome Italy; ^6^ Department of Medical Surgical Sciences and Biotechnologies Sapienza University of Rome Latina Italy; ^7^ Department of Neuroscience Catholic University of Sacred Heart Rome Italy; ^8^ Fondazione Policlinico Universitario A. Gemelli IRCCS Rome Italy; ^9^ Center for Nanotechnology Applied to Engineering (CNIS) Sapienza University of Rome Rome Italy; ^10^ Division of Gastroenterology Center for Autoimmune Liver Diseases European Reference Network on Hepatological Diseases (ERN RARE‐LIVER) IRCCS Fondazione San Gerardo Dei Tintori Monza Italy

**Keywords:** lipid nanoparticles, liver‐targeted delivery, microfluidic mixing, primary biliary cholangitis, protein corona, TIMP1 gene silencing

## Abstract

Lipid nanoparticles (LNPs) preferentially accumulate in the liver, but hepatic tropism alone does not ensure productive RNA interference. Here, we screened a library of LNPs in which lipid composition was varied to generate distinct protein corona identities and identify formulations capable of combining liver accumulation with functional siRNA delivery. LNP1 emerged as the lead formulation, showing favorable physicochemical properties, high transfection efficiency, and enhanced hepatic accumulation. Proteomic analysis associated this behavior with a distinct multicomponent corona signature. When loaded with siRNA targeting tissue inhibitor of metalloproteinases 1 (TIMP1), a key regulator of extracellular matrix turnover and fibrosis, LNP1 was efficiently internalized by primary Kupffer cells (KCs) and induced robust target knockdown. Consistent with this cellular preference, quartz crystal microbalance measurements showed that corona formation increased LNP1 binding to MARCO, a scavenger receptor expressed by KCs. In a mouse model of primary biliary cholangitis, repeated administration of siTIMP1‐loaded LNP1 reduced TIMP1 expression in KCs, reactivated extracellular‐matrix‐degradative pathways, suppressed pro‐inflammatory signaling and macrophage infiltration, alleviated cholestasis and attenuated liver fibrosis. These findings connect systematic variation in lipid composition with corona identity, hepatic tropism, and functional gene silencing, supporting protein corona engineering as a strategy to bridge liver accumulation with productive siRNA delivery.

## Graphical abstract

By shaping the protein corona, lipid composition directs LNP tropism and enables the preferential delivery of a TIMP1‐targeting siRNA to liver macrophages. In a murine model of primary biliary cholangitis, this strategy promoted macrophage reprogramming and attenuated liver fibrosis.

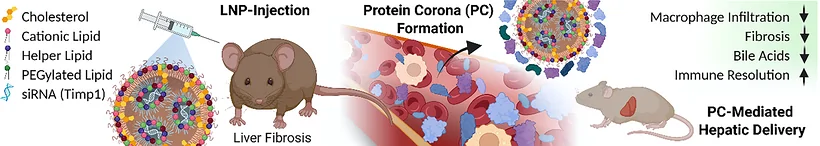

**Abbreviations**
A/G: Albumin/Globulin ratio
ALB: Albumin
ALP: Alkaline Phosphatase
ALT: Alanine Aminotransferase
AMAs: Antimitochondrial Antibodies
APO: Apolipoprotein
ARE‐Del‐/‐: Adenylate‐Uridylate–Rich Element deleted mice
AST: Aspartate Aminotransferase
CFH: Complement Factor H
CHOL: Cholesterol
DC‐Cholesterol: *3β‐[N‐(N′,N′‐dimethylaminoethane)‐carbamoyl] cholesterol hydrochloride*
DEGs: Differentially Expressed Genes
DOPE: *1,2‐Dioleoyl‐sn‐glycero‐3‐phosphoethanolamine‐N‐polysarcosine‐25*
DOPE‐PEG2k: *1,2‐Dioleoyl‐sn‐glycero‐3‐phosphoethanolamine‐N‐[amino(polyethylene glycol)‐2000]*
DOTAP: *1,2‐Dioleoyl‐3‐trimethylammonium‐propane*
DSPC: *1,2‐Distearoyl‐sn‐glycero‐3‐phosphocholine*
DSPE‐PEG2k: *1,2‐Distearoyl‐sn‐glycero‐3‐phosphoethanolamine‐N‐[carboxy(polyethylene glycol)‐2000]*
PdI: Polydispersity Index
RPA: Relative Protein Abundance

## 1 Introduction

Lipid nanoparticles are the most clinically advanced platform for nucleic acid delivery, with a well‐known propensity to accumulate in the liver after systemic administration [1, 2, 3]. However, this intrinsic tropism does not always translate into effective RNA interference, as cellular internalization often fails to result in productive cytosolic release and target gene silencing [4, 5]. Understanding the factors that govern both nanoparticle accumulation and intracellular processing remains critical for RNA therapeutics.

In this context, the protein corona (PC), the dynamic layer of plasma proteins adsorbed onto nanoparticle surfaces, is a key determinant of biological identity [6, 7, 8, 9]. Beyond shaping biodistribution, it actively regulates uptake pathways, intracellular trafficking, and endosomal escape, directly influencing functional delivery [10, 11, 12]. This recognition has shifted the field from viewing corona formation solely as an effect to be minimized toward exploiting and engineering it to direct nanoparticles to specific tissues and cell types.

Previous studies, including our own, have demonstrated that variations in cationic lipids, helper membrane lipids, and PEG‐anchoring lipids profoundly reshape nanoparticle‐proteome interactions by modulating protein corona formation [13, 14, 15]. Building upon this framework, we designed a library of four LNPs in which these structural elements were selectively varied to generate distinct corona‐mediated biological identities and ultimately identify formulations combining hepatic tropism with efficient and functional siRNA delivery.

Comparative analyses identified a lead formulation with favorable physical‐chemical properties, high transfection efficiency (TE) in vitro, and enhanced liver accumulation in vivo, featuring a corona profile associated with hepatic recognition.

When loaded with siRNA targeting tissue inhibitor of metalloproteinases 1 (TIMP1), a key regulator of extracellular matrix (ECM) turnover and fibrosis [16, 17, 18], the platform efficiently silenced targets in vitro in Kupffer cells (KCs). For in vivo analyses, we used a primary biliary cholangitis (PBC) mouse model, which recapitulates key features of fibrotic and inflammatory liver disease, the ARE‐Del‐/‐ mice, which have chronic low‐level expression of interferon‐γ (IFN‐γ) due to a deletion in the AU‐rich elements of the Ifng 3′ UTR [19]. This ongoing inflammatory signaling drives portal inflammation, bile duct injury, and fibrotic remodeling‐hallmarks of PBC [20, 21].

In this model, repeated administration of siTIMP1‐loaded LNPs selectively knocked down TIMP1, reactivated ECM‐degradative pathways, reduced pro‐inflammatory chemokines and macrophage infiltration, alleviated cholestasis, and attenuated liver fibrosis.

Our results suggest the importance of protein corona engineering as a generalizable design strategy to bridge the gap between hepatic accumulation and functional intracellular siRNA delivery, providing a broadly applicable framework for RNA‐based therapies targeting fibrotic and inflammatory liver disorders.

## 2 Results and Discussion

### 2.1 Physicochemical Characterization of Bare and Coronated LNPs

We first characterized the four pristine LNP formulations, whose lipid compositions are reported in Figure 1a–d and Table 1 of the Experimental Section, and then examined how their physicochemical identity changed upon exposure to human plasma. Incubation with excess human plasma generated protein‐coated particles, hereafter referred to as coronated LNPs, and induced measurable changes in all formulations, consistent with protein corona formation [7, 22, 23]. The systematic comparison between pristine and coronated LNPs was intended to link synthetic identity with the acquired biological identity and to provide a basis for interpreting subsequent differences in biodistribution and functional activity.

**FIGURE 1:**
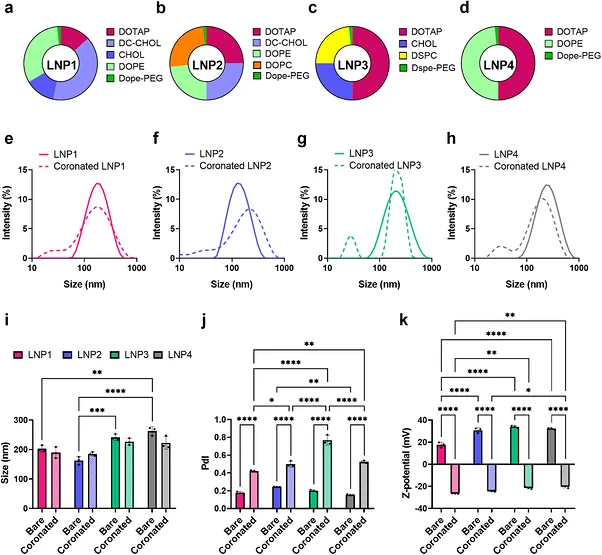
Physicochemical characterization of bare and coronated LNPs (a–d) Lipid composition of the investigated LNP formulations. (e–h) Size distribution of LNPs encapsulating pDNA (pmirGlo) before and after incubation with 50% human plasma. Physicochemical properties, including (i) size, (j) polydispersity index (PdI), and (k) Z‐potential. Statistical analysis was performed using two‐way ANOVA followed by Tukey's multiple‐comparison test. Statistical significance was defined as *p* < 0.05. Significance levels were indicated as follows: ^*^ *p* < 0.05, ^**^ *p* < 0.01, ^***^ *p* < 0.001, and ^**^ *p* < 0.0001.

**TABLE 1:** Lipid composition of LNP1‐LNP4.

	LNP1	LNP2	LNP3	LNP4
**DOTAP**	0.132	0.25	0.5	0.5
DC‐cholesterol	0.4	0.25	0	0
Cholesterol	0.133	0	0.25	0
DOPC	0	0.25	0	0
DSPC	0	0	0.235	0
DOPE	0.32	0.235	0	0.485
DOPE‐PEG2k	0.015	0.015	0	0.015
DSPE‐PEG2k	0	0	0.015	0

Size distribution profiles (Figure 1e–h) displayed peak broadening upon plasma incubation, with emerging low‐nanometer signals likely reflecting unbound plasma proteins [23]. Despite these changes, the average hydrodynamic diameter (Figure 1i) remained largely unchanged, suggesting that corona formation did not induce massive nanoparticle aggregation or fusion. However, an increase in polydispersity index (PdI) was observed after plasma exposure (Figure 1j), indicating increased colloidal heterogeneity. These findings are consistent with previous reported trends of nanoparticle‐protein interactions [24].

Zeta potential measurements (Figure 1k) revealed a shift from initially positive values ‐consistent with cationic lipid content‐ toward negative values (approximately −24 mV) after plasma exposure. This charge inversion is indicative of the adsorption of negatively charged serum proteins and is consistent with the well‐documented “surface charge normalization” phenomenon associated with PC formation [22]. Importantly, bare LNP1 and LNP2 exhibited a smaller size and also demonstrated higher colloidal stability post‐corona formation, with a smaller PdI increase in respect to bare LNP3 and LNP4.

### 2.2 Identification of Protein Corona Composition Across LNP Formulations

To define the composition of the protein corona, we performed comprehensive proteomic profiling by nano‐liquid chromatography–tandem mass spectrometry (nanoLC–MS/MS). This analysis identified the plasma proteins adsorbed onto each LNP formulation and provided a molecular basis for interpreting formulation‐dependent differences in cellular interactions and in vivo biodistribution.

Quantitative nanoLC–MS/MS analysis revealed that modulation of lipid composition markedly reshaped the protein corona, with the relative abundance of corona‐associated proteins grouped by functional category and visualized as heatmaps in Figure 2.

**FIGURE 2:**
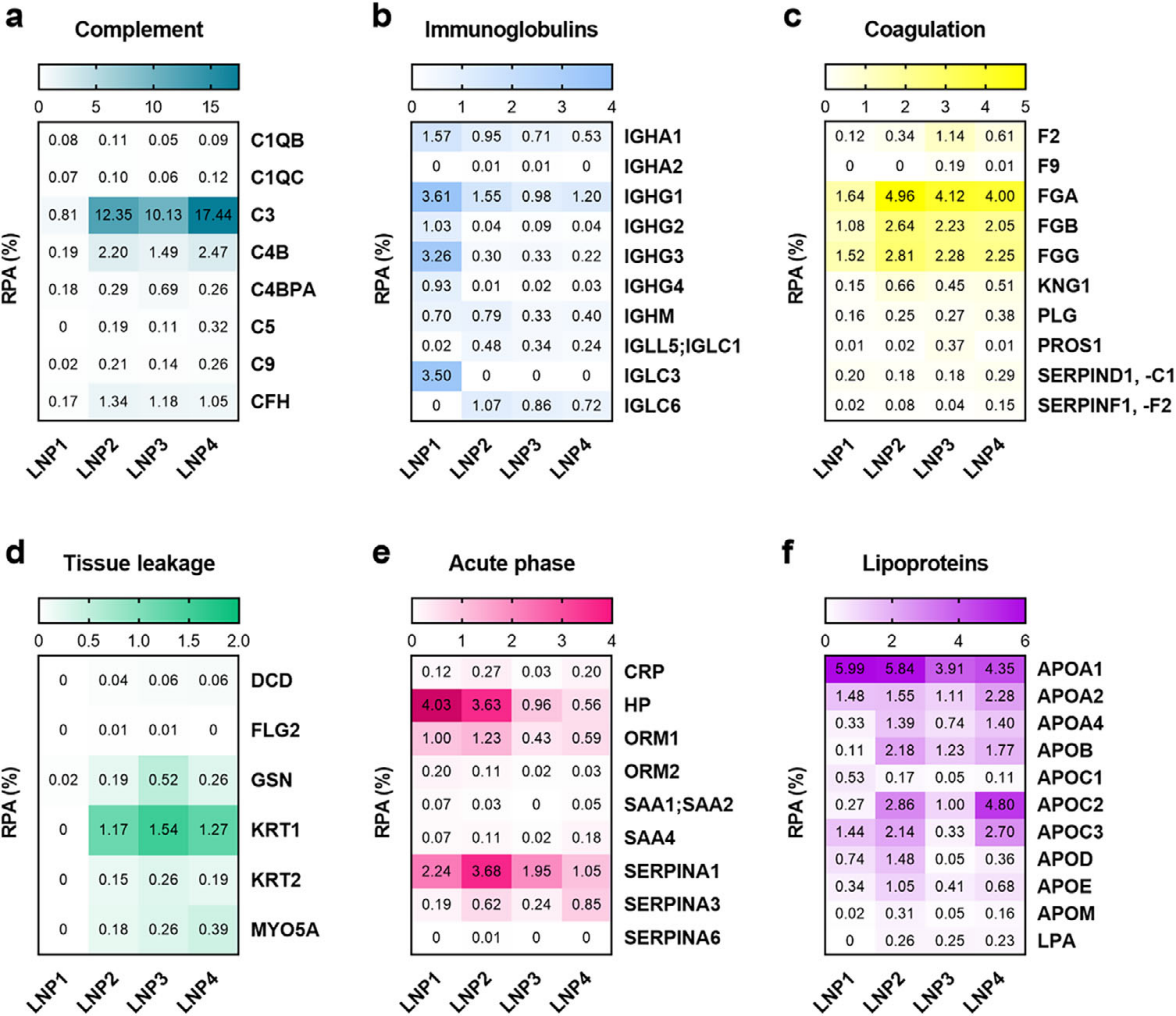
Functional classification of protein corona composition across LNP formulations. (a–f) Colormap of RPA for each protein of any functional category. Proteins with an RPA lower than 0.1% were not displayed in the graphical representation. The full protein list is provided in Table S1.

Rather than producing minor quantitative fluctuations, each formulation acquired a distinct proteomic profile, demonstrating that variations in cationic lipids, helper lipids, and PEG‐anchoring lipids effectively reprogram nanoparticle‐proteome interactions and generate different biological identities.

To contextualize these differences, we briefly highlight the main formulation‐dependent trends across functional protein categories. These observations should not, however, be interpreted as direct evidence of biological function, as protein abundance alone does not account for factors such as adsorption orientation, structural accessibility, or retention of receptor‐binding activity within the corona [25, 26, 27].

Within the complement category (Figure 2a), C3 was strongly enriched in LNP4 and progressively lower in LNP2, LNP3, and LNP1, where it was nearly absent. C4B and complement factor H followed the same trend at lower abundance. As complement proteins promote opsonization and immune recognition [28], their enrichment may favor clearance by the mononuclear phagocyte system [29, 30].

Immunoglobulins showed the opposite pattern (Figure 2b), being predominantly associated with LNP1. IGLC3 was detected only in LNP1, together with higher levels of IGHA1, IGHG1, and IGHG3. Their effect on immune recognition may depend on adsorption orientation within the corona [30].

Coagulation proteins were comparatively enriched in LNP2–4 and depleted in LNP1 (Figure 2c). Fibrinogen chains were consistently more abundant in LNP2–4, whereas prothrombin was highest in LNP3. Together with complement and immunoglobulins, these proteins may contribute to opsonization and clearance [31]. Tissue leakage proteins, including KRT1, MYO5, and GSN, were detected at low levels in LNP2–4 but were absent or nearly undetectable in LNP1 (Figure 2d), a pattern potentially relevant to recognition by the mononuclear phagocyte system [32].

Among acute‐phase proteins (Figure 2e), haptoglobin was enriched in LNP1 and LNP2, whereas Serpina1 was highest in LNP2. These proteins may differently influence macrophage recognition and inflammatory responses [33, 34].

LNP1 and LNP2 showed the most prominent apolipoprotein enrichment (Figure 2f), with high APOA1 levels and selective enrichment of APOC1 and APOD in LNP1 and a broader apolipoprotein profile in LNP2. By contrast, albumin was most abundant in LNP1, whereas vitronectin showed the opposite trend and was strongly enriched in LNP3 and LNP4 (Figure S1).

Although no one‐to‐one relationship emerged between individual lipid species and single plasma proteins, variations in the lipid interface were associated with coordinated remodelling of functionally related protein families. Formulations containing cationic DC‐cholesterol [35] (LNP1 and LNP2) preferentially recruited coronas enriched in APOA1, APOD and haptoglobin and depleted in vitronectin, whereas formulations lacking DC‐cholesterol (LNP3 and LNP4) displayed a shift toward complement‐, coagulation‐ and vitronectin‐rich coronas. These findings suggest that lipid composition modulates the biological identity of LNPs primarily through coordinated changes in multicomponent protein networks rather than through selective recruitment of individual proteins. Such formulation‐dependent differences in protein corona composition are therefore expected to influence immune recognition, biodistribution, and overall in vivo fate. Building on this observation, we next evaluated the ability of the LNPs to deliver nucleic acid payloads in vitro and investigated their biodistribution in vivo.

### 2.3 Functional Validation In Vitro and Organ‐Specific Biodistribution In Vivo

To compare the transfection performance of the LNP formulations and assess how the protein corona influences their interaction with cells, we encapsulated a firefly luciferase‐encoding plasmid DNA (pDNA) and tested the resulting particles in HEK‐293 cells (Figure 3a). HEK‐293 cells were used as an initial benchmark [36] because of their high transfectability and robust expression of exogenous genes, enabling a clear comparison among formulations before extending the analysis to more challenging cell models, including RAW 264.7 macrophages.

**FIGURE 3:**
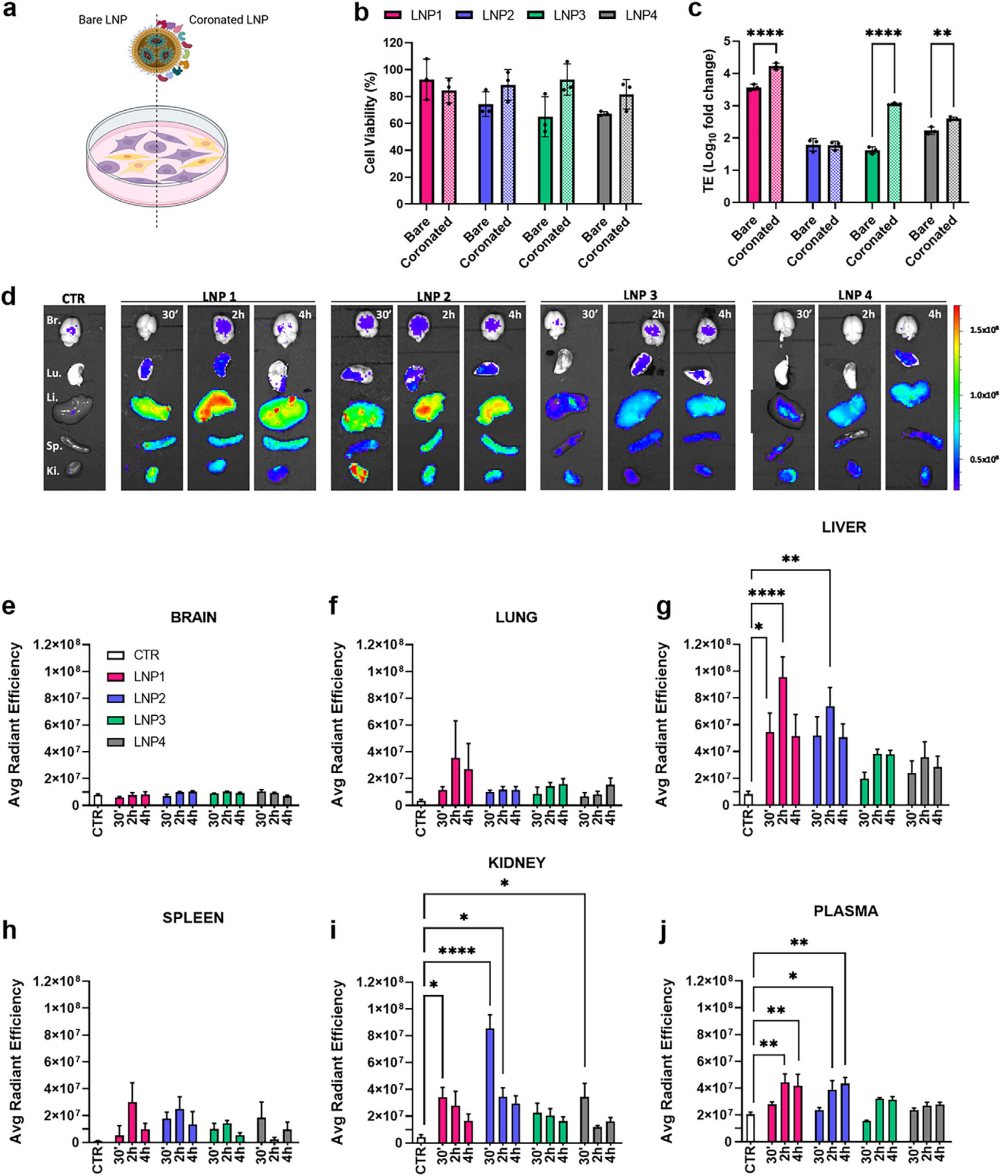
In vitro validation of LNPs and ex vivo time‐course biodistribution. (a) Representative sketch for the in vitro experiments made using bare and coronated LNPs (b) Cell viability (percentage of viable cells compared with control, untreated cells) after treatment of HEK‐293 cells using LNPs encapsulating pDNA coding for a reporter gene (firefly luciferase) before and after incubation with 50% human plasma volume, and (c) TE expressed as Log10 of the Fold change. (d) Representative *ex vivo* IVIS fluorescence imaging of harvested organs (Brain, Br.; Lung, Lu.; Liver, Li.; Spleen, Sp.; Kidney, Ki.) at 30 min, 2 h, and 4 h post‐intraperitoneal injection of Texas Red‐labeled LNPs in healthy mice compared to untreated controls (CTR). The pseudo‐color scale bar represents the fluorescence signal intensity, measured as Radiant Efficiency (p/s/cm^2^/sr)/(µW/cm^2^). (e–j) Quantitative analysis of organ‐specific accumulation and plasma circulation kinetics expressed as Average Radiant Efficiency across distinct organs: (e) Brain, (f) Lung, (g) Liver, (h) Spleen, (i) Kidney, and (j) Plasma. Statistical significance was assessed using two‐way ANOVA with Tukey's multiple comparisons. Differences were considered statistically significant at *p* < 0.05 and are indicated as follows: ^*^ *p* < 0.05, ^**^ *p* < 0.01, ^***^ *p* < 0.001, and ^**^ *p* < 0.0001.

As shown in Figure 3b, LNP1 and LNP2 were well tolerated in their pristine form and retained high cell viability after plasma preincubation. By contrast, pristine LNP3 and LNP4 showed mild cytotoxicity, which was attenuated following protein corona formation. Overall, plasma exposure improved biocompatibility, consistent with reports that adsorbed proteins can attenuate nanoparticle‐associated cytotoxicity by reducing surface charge and masking immunogenic components [37].

As shown in Figure 3c, protein corona formation did not impair transfection efficiency in any formulation. Instead, it enhanced transfection for LNP1, LNP3, and LNP4, while leaving LNP2 largely unchanged. LNP1 consistently achieved the highest activity across conditions. These findings indicate that plasma adsorption can preserve or improve LNP function and that specific corona profiles may promote productive transfection [38].

To assess the in vivo biodistribution of the four formulations, LNPs were labelled with Texas Red and administered intraperitoneally to healthy mice. Organ‐specific accumulation was then quantified ex vivo by IVIS imaging of harvested tissues at 30 min, 2 h, and 4 h after injection (Figure 3d).

Quantification of the radiant efficiency confirmed distinct organ tropism across the formulations (Figure 3e–j). For all LNPs, no signal accumulation was found in the brain (Figure 3e), while lung and spleen uptake was low and transient (Figure 3f,h), suggesting successful avoidance of the splenic mononuclear phagocyte system. Conversely, prominent differences were observed in the liver (target tissue), and in the kidneys, key organs mainly devoted to clearance. In the liver, LNP1 and LNP2 demonstrated significantly higher tropism compared to LNP3 and LNP4, peaking at 2 h (Figure 3g).

Interestingly, the two formulations exhibiting the highest hepatic accumulation (LNP1 and LNP2) converged toward a shared protein corona phenotype despite differences in the relative abundance of several individual proteins. Rather than being defined by a single dominant ligand, this phenotype was characterized by the coordinated enrichment of APOA1, haptoglobin, and albumin, together with a relative depletion of vitronectin, compared with the less hepatotropic formulations (LNP3 and LNP4). From a physiological perspective, these proteins are all intimately connected with liver function. APOA1 is the major structural protein of high‐density lipoproteins (HDL), which mediate reverse cholesterol transport by delivering peripheral cholesterol to the liver through interactions with hepatocyte‐expressed scavenger receptor class B type I (SR‐BI) [39, 40]. Albumin, the most abundant plasma protein, is synthesized predominantly by hepatocytes and is responsible for maintaining plasma oncotic pressure while serving as the principal carrier of endogenous metabolites and numerous circulating molecules [41]. Haptoglobin is likewise produced predominantly by the liver as a major acute‐phase protein and contributes to hemoglobin clearance through the CD163 scavenger pathway in tissue macrophages, including Kupffer cells [33]. Conversely, vitronectin is an adhesive glycoprotein present in plasma and the extracellular matrix, where it contributes to cell adhesion, complement regulation, and integrin‐mediated recognition [42], and has previously been implicated in alternative organ‐targeting pathways in nanoparticle systems [43]. Although none of these proteins can individually be regarded as a mechanistic determinant of hepatic uptake, their coordinated enrichment delineates a biologically coherent corona phenotype associated with enhanced liver accumulation. Importantly, this interpretation differs substantially from reductionist models in which the biological fate of nanoparticles is attributed to a single adsorbed protein. Our findings instead support the view that hepatic tropism emerges from the collective biological identity encoded within the protein corona. In this framework, the corona should be regarded as an emergent supramolecular entity whose function cannot be inferred solely from the abundance of its individual constituents.

At present, the most reliable conclusion supported by our data is that modulation of lipid composition generates distinct protein corona phenotypes, which are in turn associated with distinct in vivo biodistribution profiles. Our proteomic analysis also prompts a broader reconsideration of the current interpretation of protein corona‐mediated liver targeting. ApoE has been widely regarded as one of the principal mediators of endogenous hepatic delivery of lipid nanoparticles [1, 44, 45, 46, 47]. However, in the present study, its relative abundance did not parallel the biodistribution profile of the four formulations. Notably, LNP1, which exhibited the most pronounced hepatic accumulation, contained one of the lowest relative amounts of ApoE within its protein corona, whereas LNP2 displayed the highest ApoE abundance without a corresponding increase in liver tropism. Moreover, ApoE represented only a minor constituent of all four coronas, accounting for a small fraction of the adsorbed proteome. These observations do not exclude a functional role for ApoE but rather suggest that its abundance alone is insufficient to explain the biological fate of lipid nanoparticles. More fundamentally, nanoLC‐MS/MS provides quantitative information on protein abundance but does not reveal the supramolecular organization of the protein corona. The spatial localization, orientation, conformational state, and surface accessibility of individual proteins and therefore the actual availability of their receptor‐binding epitopes remain largely unknown. Therefore, assigning complex biological functions to individual corona constituents based on abundance alone may lead to an oversimplified interpretation of what is intrinsically an emergent biological interface [48]. Conversely, our data consistently support a more conservative conclusion: changes in lipid composition generate distinct protein coronas, and distinct protein coronas are associated with different in vivo biodistribution profiles. Within this framework, the protein corona should be regarded primarily as an emergent systems‐level determinant of biological identity, whereas the contribution of individual proteins is more appropriately investigated a posteriori, once a functionally relevant corona has been identified and after experimentally establishing the exposure and biological accessibility of specific surface epitopes. Addressing these mechanistic questions will require dedicated structural and receptor‐level studies that extend well beyond the scope of the present work.

Interestingly, LNP2 showed a massive, transient accumulation in the kidneys at 30 min post‐injection, which rapidly dropped by the 2‐h mark (Figure 3i). This observation likely indicates LNP2 instability upon systemic administration, leading to formulation fragmentation and subsequent renal clearance of smaller components. Lastly, signal tracking in plasma revealed a steady presence of circulating nanoparticles, in particular LNP1 and LNP2 up to 4 h (Figure 3j).

To orthogonally validate these circulation kinetics, a detailed spectrofluorometric analysis of plasma was conducted, covering an emission spectrum from 585 to 650 nm (Figure S2a–d). Consistent with the whole‐organ imaging, plasma from mice treated with LNP1 and LNP2 displayed a strong, time‐dependent fluorescence emission profile peaking around the characteristic wavelength of Texas Red, while LNP4 showed negligible signal over background levels. Direct quantification of the fluorescence intensity at the peak emission (610 nm) confirmed significant plasma retention for LNP2 at both 2 and 4 h, followed closely by LNP1 at 4 h (Figure S2e).

Taken together, these data demonstrate that both LNP1 and LNP2 formulations have excellent pharmacokinetic profiles. However, a critical divergence was observed in their organ tropism and clearance pathways. LNP2 showed a large transient trapping in the kidneys at 30 min (Figure 3i), suggesting rapid renal clearance or non‐specific renal uptake, LNP1 maintained low, controlled renal levels while showing a highly sustained and progressive accumulation specifically in the liver (Figure 3g).

Therefore, LNP1 was clearly identified as the optimal formulation for all subsequent therapeutic silencing and efficacy studies in the chronic fibrotic mouse model, owing to its better hepatic targeting profile and significantly better in vitro TE.

Because the subsequent therapeutic studies were designed to target KCs, we selected MARCO as a biologically relevant receptor for mechanistic validation. MARCO is a class A scavenger receptor expressed by KCs, with extensive evidence supporting its involvement in the recognition and clearance of circulating particulate material [49, 50, 51]. We therefore asked whether the biological identity acquired by LNP1 after plasma exposure enhanced its interaction with this receptor. Using quartz crystal microbalance measurements (QCM) on MARCO‐functionalized sensor chips, we compared binding of pristine and coronated LNP1. The mass deposition on the functionalized sensor surfaces was calculated from frequency shifts as explained in the Experimental Section. MARCO‐functionalized QCM wafer exhibited a robust and rapid mass increase upon the injection of coronated LNP1, reaching approximately 896 ng within 2 h (Figure S3). Injection of bare LNPs resulted in a negligible mass change (60 ng), confirming that the LNPs do not exhibit significant non‐specific binding to the MARCO‐coated surface in the absence of the corona. This indicates that protein corona formation creates a biological interface with increased affinity for MARCO [52]. These findings support preferential recognition of coronated LNP1 by MARCO‐expressing KCs and provide a mechanistic rationale for its subsequent functional evaluation in this cell population.

Finally, to evaluate LNP1 intrahepatic localization, *ex vivo* fluorescence microscopy was performed on liver sections from healthy treated mice stained with CD68 antibody, given the therapeutic interest in targeting KCs (Figure S4). Texas Red‐labeled LNP1 rapidly accumulated in a diffuse punctate pattern at 30 min, intensifying at 2 and 4 h, confirming sustained hepatic retention and close interface contact with CD68+ macrophages. Mechanistically, Triton‐X‐100 was omitted to prevent LNP lipid disruption, restricting antibody binding to the small fraction of CD68 physiologically expressed on the macrophage plasma membrane rather than the internal lysosomal pool [53]. This technical configuration accounts for the apparent lack of chromatic colocalization (yellow merge signal) despite clear spatial engagement.

### 2.4 Selection of Fibrosis‐Associated Key Regulatory Genes as Therapeutic Targets

To identify therapeutic targets relevant to liver fibrosis and specifically associated with macrophage function, we performed an integrative analysis of two transcriptomic datasets: GSE76309 (whole liver RNAseq from ARE‐Del‐/‐ mice) and GSE226946 (bulk RNA‐seq of KCs from CCl_4_‐induced fibrotic livers). We performed differential gene expression analysis in RStudio, focusing first on genes that were significantly downregulated in both datasets, to map the fibrotic liver transcriptome related to the KC‐specific profile. Pathway enrichment analysis with Metascape [54] revealed that these overlapping downregulated genes were involved in ECM organization, angiogenesis, MAPK signaling, and cell migration, processes commonly impaired during chronic liver fibrosis (Table S2).

Next, we examined genes upregulated in ARE‐Del‐/‐ mice, prioritizing those linked to the aforementioned fibrotic pathways (Figure S5 and Table S3). Among them, TIMP1 emerged as a valid candidate, due to its high expression in our PBC mice model and its dual role in modulating both fibrotic and inflammatory processes [55]. TIMP1 acts as an inhibitor of matrix metalloproteinases, particularly in chronic liver injury, and its activity inhibits MMP9. This process often leads to pathological ECM accumulation, increased tissue stiffness, and sustained hepatic stellate cell activation [16]. In addition to inhibiting MMPs, TIMP1 exhibits a cytokine‐like action, influencing hepatocyte apoptosis, proliferation, and angiogenesis [17]. Considering its central role in maintaining the fibrotic microenvironment, together with its higher expression in the livers of PBC mice, TIMP1 was selected to be silenced by loading a specific siRNA in LNP1. To achieve this objective, the lipid formulation of LNP1 was employed to encapsulate siTIMP1. Characterization of siTIMP1‐LNPs revealed a slightly larger size relative to pDNA‐loaded LNP1 (≈250 nm vs. ≈200 nm; Figure S6), highlighting the structural role of the encapsulated nucleic acid.

### 2.5 siTIMP1‐LNPs Efficiently Deliver siRNA for TIMP1 to Liver Macrophages In Vitro and In Vivo

The selectivity and specificity of siTIMP1‐LNPs were further investigated in a third series of in vitro experiments using LPS‐stimulated RAW264.7 macrophages, a well‐established model of inflammation‐induced Timp1 upregulation [18]. Treatment with siTIMP1‐LNPs induced a dose‐dependent reduction of Timp1 mRNA levels after 24 h, compared to scrambled siRNA controls (Figure 4a). However, RAW264.7 cells, as an immortalized murine macrophage‐like line, exhibit distinct features compared to primary tissue‐resident macrophages, including altered receptor profiles and exaggerated inflammatory responses [56]. To validate the biological relevance of our findings in a more physiologically related setting, we tested siTIMP1‐LNPs in primary KCs isolated from the liver of ARE‐Del‐/‐ mice, where both 0.125 and 0.5 nmol doses of siTIMP1‐LNPs significantly downregulated Timp1 expression (Figure 4b). To assess the functional relevance of TIMP1 silencing, we quantified Mmp9 expression, a key ECM‐degrading enzyme regulated by TIMP1. Both RAW264.7 macrophages and primary KCs showed a dose‐dependent upregulation of Mmp9 following siTIMP1‐LNP treatment (Figure 4c,d), indicating reactivation of ECM‐remodeling pathways.

**FIGURE 4:**
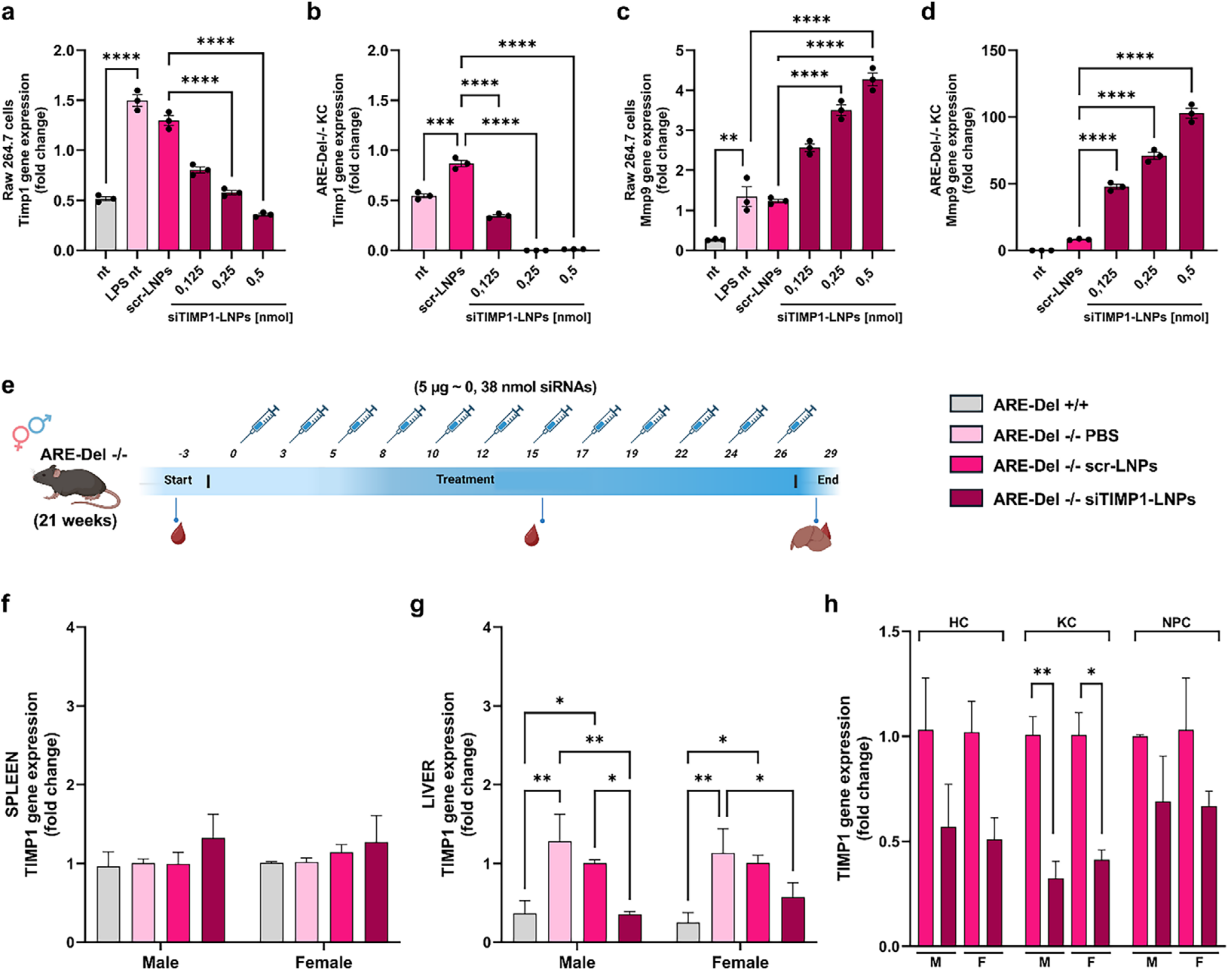
siTIMP1‐loaded LNPs selectively silence Timp1 in liver macrophages and restore ECM‐degrading pathways. (a) Dose‐dependent knockdown of Timp1 in LPS‐stimulated RAW264.7 macrophages treated for 24 h with siTIMP1‐LNPs (0.125–0.5 nmol). (b) Validation in primary KCs isolated from ARE‐Del‐/‐ mice shows a significant reduction of Timp1. (c,d) Functional rescue of Mmp9 expression following Timp1 silencing in macrophages upon siTIMP1‐LNP treatment. (e) Schematic representation of the in vivo treatment protocol: 21‐week‐old ARE‐Del‐/‐ mice received 12 serial i.p. injections of siTIMP1‐LNP (5 µg/injection) between Day 0 and Day 26, after the baseline blood sampling at Day ‐3, with endpoint sacrifice at Day 29 (created in BioRender). (f–g) Quantitative RT‐PCR analysis of the whole spleen and liver confirms significant Timp1 mRNA reduction. (h) Transcriptional analysis of isolated liver cell populations (HCs, KCs, and NPCs) from male (M) and female (F) mice, reveals selective Timp1 knockdown in KCs. Data are presented as mean ± SEM. For in vitro assays, *n* = 3 independent wells were analyzed for RAW 264.7 cells, and primary KCs were obtained from *n* = 3 independent mouse extractions. For in vivo experiments, *n* = 3 animals per group were used. Statistical significance was determined using one‐way or two‐way ANOVA followed by Sídák's multiple comparisons test (^*^ *p* < 0.05, ^**^ *p* < 0.01, ^***^ *p* < 0.001, and ^****^ *p* < 0.0001).

This body of evidence encouraged us to translate our findings from in vitro to in vivo validation. It is well known that the functional efficacy of many nucleic acid‐based therapies requiring intracellular delivery often declines significantly when tested in animal systems, a limitation commonly attributed to physiological barriers, immune surveillance, and the instability of nucleic acids in systemic circulation [57]. To overcome these challenges and evaluate chronic therapeutic silencing, 21‐week‐old ARE‐Del‐/‐ mice of both sexes were subjected to a 5‐week experimental protocol. As schematically illustrated in the experimental timeline (Figure 4e), following baseline tracking of body weight and blood collection at Day ‐3, mice received a total of 12 serial i.p. injections of siTIMP1‐LNPs (1.23 mg kg^−1^, corresponding to 5 µg siRNA or 0.38 nmol of siRNA per injection) administered at days 0, 3, 5, 8, 10, 12, 15, 17, 19, 22, 24, and 26. The decision to include both sexes at the same age stems from a series of previous studies [20] showing that disease onset, clinical progression, and incidence differ significantly between males and females, with an earlier, higher, and more aggressive form observed in females.

At the end of the treatment window (Day 29), TIMP1 mRNA levels measured in treated animals confirmed the validity of our approach. Livers from mice treated with siTIMP1‐LNPs showed a marked reduction in TIMP1 expression compared to vehicle‐treated controls, with no significant differences observed between sexes (Figure 4g), while no silencing was detected in the spleen (Figure 4f), supporting tissue selectivity. To determine cell‐type specificity, we isolated hepatocytes (HCs), KCs, and non‐parenchymal cells (NPCs) from treated livers. Efficient Timp1 knockdown (∼70% in males and ∼50% in females) was observed exclusively in KCs, with no significant reduction in HCs or NPCs, confirming selective targeting of hepatic macrophages by the siTIMP1‐LNP formulation. (Figure 4h). These findings demonstrate the potential of siTIMP1‐LNPs to selectively target hepatic macrophages, enhancing ECM remodeling and offering a promising therapeutic avenue for liver fibrosis in PBC.

### 2.6 TIMP1 Silencing Restores MMP9 Expression and Preserves ECM Remodeling Machinery

Silencing Timp1 was a necessary step to validate our strategies; however, additional downstream effects needed to be investigated to demonstrate the biological reliability and translational potential of our system. First, we investigated whether chronic siTIMP1‐LNP administration could intensify ECM‐degrading activity by quantifying the expression of the gelatinase Mmp9. As expected, siTIMP1‐LNP significantly increased the Mmp9 transcript levels in male and female ARE‐Del‐/‐ mice compared to vehicles and scr‐LNP‐treated mice (Figure 5a). Notably, the treatment with scr‐LNP did not induce Mmp9 upregulation, further confirming that the observed effect is specifically mediated by Timp1 knockdown and excluding nonspecific transcriptional responses due to nanoparticle accumulation, which may otherwise cause transient metabolic impairment unrelated to defined molecular pathways. The induction of Mmp9 was particularly evident in KC (Figure 5b), confirming their role as the primary cellular target of the formulation and a key effector population for ECM remodeling. These findings are consistent with prior evidence showing that KC‐derived MMP9 contributes to fibrosis resolution by promoting collagen degradation and matrix turnover [58].

**FIGURE 5:**
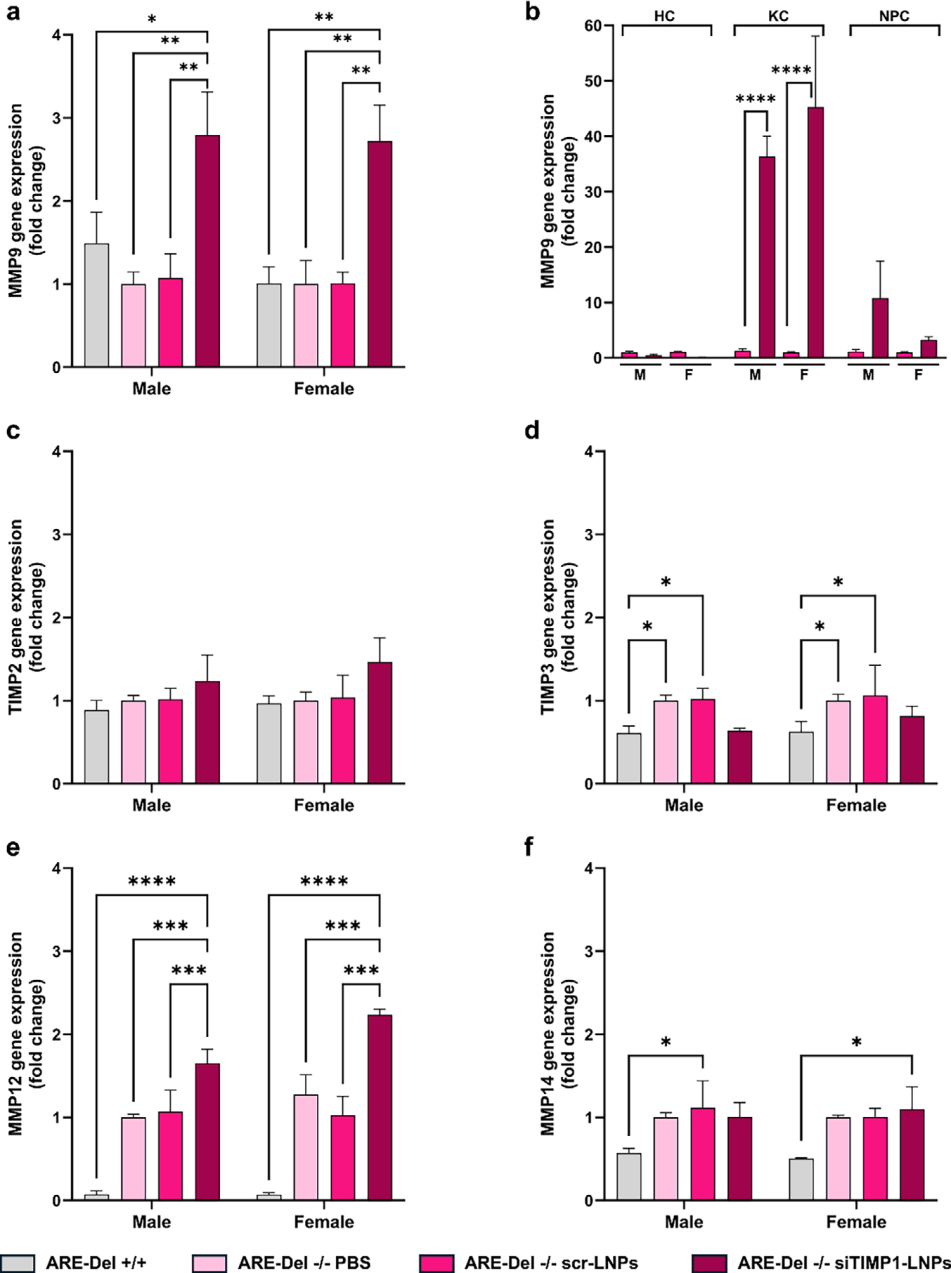
TIMP1 silencing reactivates Mmp9 expression and preserves the ECM remodeling program. (a) Mmp9 expression is significantly upregulated in the total liver of male and female ARE‐Del‐/‐ mice following siTIMP1‐LNP treatment compared to PBS and scrambled control (scr‐LNPs). (b) Cell‐type‐specific transcriptional analysis in isolated liver populations shows selective Mmp9 induction in KCs of both male (M) and female (F) mice, but not in HCs or NPCs. (c,d) Expression levels of Timp2 (c) and Timp3 (d) in whole liver. Endogenous levels remain unaffected by treatment, indicating the absence of off‐target effects. (e) Hepatic expression of Mmp12, showing a significant increase induced in both sexes after siTIMP1‐LNP administration, suggesting downstream reactivation of additional ECM‐degradative enzymes. (f) Transcriptional levels of Mmp14 across treatment groups, showing no major changes following treatment, further supporting the specificity of the siTIMP1‐LNP formulation. Data are presented as mean ± SEM (*n* = 3 animals per group). Statistical significance was determined by two‐way ANOVA followed by Sídák's multiple comparisons test (^*^ *p* < 0.05, ^**^ *p* < 0.01, ^***^ *p* < 0.001, and ^****^ *p* < 0.0001).

Since TIMP1 belongs to a small family of endogenous MMP inhibitors, we next examined whether its silencing would elicit compensatory transcriptional responses involving other TIMPs. In particular, we focused on Timp2 and Timp3, which are implicated in matrix regulation and have been described as context‐dependent modulators in various fibrotic and neoplastic conditions [59]. Timp2, the most abundantly expressed member of the TIMP family, plays multiple roles beyond MMP inhibition, including regulation of vascular integrity and tumor behavior [60]. While Timp3 is often silenced via promoter methylation in tumors, it regulates inflammation and ECM turnover [59]. In cardiac dysfunction, their deficiency results in distinct remodeling phenotypes: Timp2 loss promotes hypertrophy without fibrosis, whereas Timp3 deficiency leads to fibrosis without hypertrophy [61].

In our model, however, Timp2 transcript levels remained unchanged across all treatment groups (Figure 5c), suggesting that TIMP1 silencing does not induce a broader transcriptional compensation within the TIMP family. Interestingly, Timp3 expression was significantly elevated in ARE‐Del‐/‐ mice compared to wild‐type controls, confirming its involvement in the fibrotic transcriptional program. However, Timp3 levels were not affected by siTIMP1‐LNP treatment (Figure 5d), suggesting that, although active in the disease state, Timp3 does not act as a compensatory regulator following Timp1 silencing. This reinforces the concept that TIMPs exert non‐redundant roles in vivo. In particular, TIMP1 is known to have unique pleiotropic and immunomodulatory functions, acting as a key driver of tissue remodeling and chronic inflammatory responses distinct from other members of its family [59].

We also analyzed Mmp14 (also known as MT1‐MMP), a membrane‐anchored collagenase involved in pericellular ECM degradation, hepatic stellate cell (HSC) activation, and pro‐MMP2 processing. Although MMP14 is a known low interactor of TIMP1 and its expression has been associated with tissue remodeling, angiogenesis, and immune infiltration in both fibrotic and neoplastic settings [62, 63, 64], its transcript levels remained unchanged across all treatment groups (Figure 5f), suggesting that TIMP1 silencing does not induce a generalized activation of stromal remodeling response. Rather, it promotes a selective, cell‐specific molecular response, aligning with KC‐targeted delivery and the observed antifibrotic phenotype.

In contrast, we found that Mmp12, another TIMP1‐regulated metalloproteinase [62], was consistently upregulated following treatment in both sexes (Figure 5e), supporting a coordinated reactivation of ECM‐degradative enzymes downstream of Timp1 knockdown.

These results indicate that siRNA‐mediated knockdown of Timp1 in hepatic macrophages restores Mmp9 and Mmp12 expression, promoting the ECM remodeling machinery without broadly altering the balance of matrix regulators. From a pharmacological and translational perspective, the selective reactivation of the ECM remodeling program within macrophages underscores the potential of siTIMP1‐LNPs as a targeted nanomedicine strategy to reverse fibrosis while preserving systemic matrix homeostasis.

### 2.7 siTIMP1‐LNP Treatment Reprograms the Hepatic Immune Microenvironment Toward a Regulatory and Resolving Profile

The demonstration that siRNA‐mediated Timp1 silencing via systemic LNP administration selectively activates a cascade of transcriptional events in ARE‐Del‐/‐ mice provides promising support for the therapeutic potential of this strategy. Our data revealed that the hepatic expression of Il‐10 and Il‐6 was significantly increased in siTIMP1‐LNP‐treated females compared to the other experimental groups (Figure 6a,b), indicating activation of anti‐inflammatory signaling pathways. Interestingly, although the expression of Timp1, Mmp9, and Mmp12 showed a similar trend in both sexes, here we found that the two cytokines were induced exclusively in females. This difference may reflect the more advanced and severe stage of disease typically observed in female ARE‐Del‐/‐ mice, potentially resulting in a heightened responsiveness to treatment.

**FIGURE 6:**
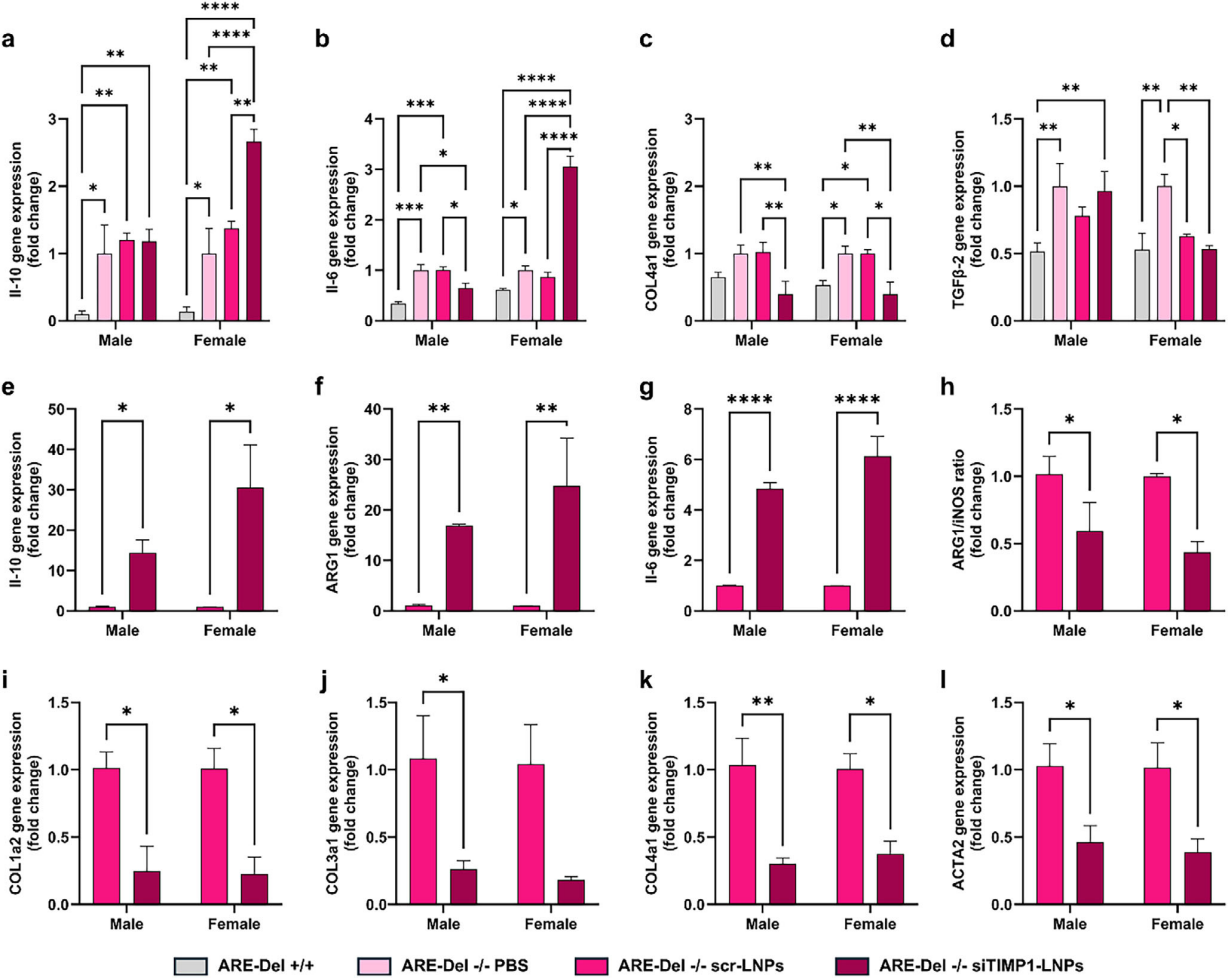
siTIMP1‐LNP treatment reprograms the hepatic immune microenvironment and suppresses fibrogenic gene expression. (a,b) qPCR analysis of total liver tissue reveals increased expression of Il‐10 (a) and Il‐6 (b) in siTIMP1‐LNP‐treated mice, indicating enhanced anti‐inflammatory responses. (c,d) Hepatic expression of the fibrogenic genes (c) Col4a1 and (d) Tgfβ2, showing significant downregulation following treatment and suggesting antifibrotic transcriptional remodeling. (e,f) Transcriptional analysis in isolated KCs demonstrates that siTIMP1‐LNP treatment induces Il‐10 (e) and Arg1 (f), suggesting a macrophage polarization toward a regulatory phenotype. (g) Transcriptional expression of Il‐6 in isolated HCs, showing a significant increase upon siTIMP1‐LNP administration and suggesting parenchymal‐to‐immune cellular cross‐talk. (h) The Arg1/iNos expression ratio in isolated NPCs, demonstrating a significant decrease following siTIMP1‐LNP treatment, reflecting complex microenvironmental transcriptional remodeling within the non‐parenchymal compartment. (i–l) mRNA expression levels of key ECM components and activation markers, including Col1a2 (i), Col3a1 (j), Col4a1 (k), and Acta2 (l) in isolated NPCs, showing generalized suppression and pointing to the deactivation of ECM‐producing fibrogenic cells. Data are presented as mean ± SEM (*n* = 3 animals per group). Statistical significance was determined by two‐way ANOVA followed by Sídák's multiple comparisons test (^*^ *p* < 0.05, ^**^ *p* < 0.01, ^***^ *p* < 0.001, and ^****^ *p* < 0.0001).

To decipher the cellular contributions to this tissue‐level response, we characterized isolated liver cell populations. In isolated KCs from siTIMP1‐LNP‐treated mice, we saw a strong induction of Il‐10 and Arg1 expression (Figure 6e,f), indicating a polarization toward a regulatory phenotype. Interestingly, Il‐6 expression was significantly upregulated in isolated HCs (Figure 6g), despite being downregulated in siTIMP1‐treated KCs in vitro (data not shown), suggesting that hepatocytes may respond secondarily to macrophage‐derived signals in vivo. This finding supports the existence of a crosstalk mechanism between parenchymal and immune compartments contributing to the overall immunomodulatory effect of TIMP1 silencing.

Since the clinical progression of PBC involves a delicate balance between the pro‐inflammatory and pro‐fibrotic phenotypes of hepatic macrophages, we next investigated whether silencing Timp1 also affected the main markers of fibrosis. In the entire liver, Tgfβ2, a key pro‐fibrotic cytokine, was significantly downregulated in females treated with siTIMP1‐LNP (Figure 6d), while Col4a1 was reduced in both sexes (Figure 6c). To further explore the antifibrotic signature, we evaluated additional ECM‐related genes in NPCs. The expression of Col1a2, Col3a1, Col4a1, and Acta2 was consistently decreased after treatment (Figure 6i–l), supporting the suppression of stromal fibrogenic activity. Concurrently, the total NPC compartment exhibited a significant decrease in the Arg1/iNos expression ratio (Figure 6h). While purified KCs adapted a clear regulatory profile, this transcriptional shift within the heterogeneous NPC fraction potentially reflects a classic pro‐inflammatory adjustment of the hepatic microenvironment. The reduced ratio may therefore indicate complex multicellular microenvironmental remodeling within the resolving fibrotic niche. These effects were consistently observed in both sexes. Overall, these data suggest that TIMP1 silencing not only affects ECM but may also contribute to a broader cellular reprogramming within the hepatic microenvironment potentially influencing chronic fibrogenesis and inflammation.

### 2.8 siTIMP1‐LNP Treatment Alleviates Cholestasis, Attenuates Macrophage Recruitment, and Reduces Hepatic Fibrosis

In the final phase of our study, we evaluated the impact of chronic siTIMP1‐LNP administration on the clinical and pathological progression of liver disease in male and female ARE‐Del‐/‐ mice, monitoring key parameters such as systemic and hepatic indicators of liver function, immune remodeling, and fibrosis. Correlation analysis revealed that Timp1 expression is embedded within a coordinated transcriptional network of fibrosis‐, inflammation‐, immune‐, and ECM‐related genes in liver tissue, segregating into three distinct co‐regulated clusters (Figure S7). Chronic treatment was well tolerated in both sexes, as shown by the absence of significant differences in body weight and viability across groups (Figure S8a,b), and by the consistent welfare scores monitored throughout the study (see Table S6). Notably, a significant reduction in liver index was observed in females treated with siTIMP1‐LNPs, with a similar trend in males (Figure S8b), suggesting a potential decrease in hepatomegaly, possibly due to the attenuation of fibrosis or inflammatory cell infiltration. The spleen index remained unchanged, indicating the absence of systemic splenomegaly or overt immune activation. Biochemical assessment of hepatic synthetic function revealed preserved serum albumin levels and a significant reduction in serum globulin concentrations, particularly in ARE‐Del‐/‐ males (Figure S8c), consistent with systemic inflammation, compared with WT animals. Moreover, serum albumin and transaminases (AST, ALT) remained stable across all groups (Figure S8e), indicating preserved hepatocellular function.

Indicators of cholestasis and lipid metabolism were positively affected by TIMP1 silencing. Specifically, TBA levels, which are extremely elevated in animals with cholestatic liver injury, were significantly reduced compared to vehicle and scr controls (Figure 7a), indicating improved bile acid clearance. Hepatic Timp1 expression showed a positive correlation with circulating TBA (r = 0.6355, p = 0.0011; Figure 7b), reinforcing the idea of a mechanistic link between TIMP1 and bile acid accumulation. This aligns with the established pathophysiology of PBC, where impaired bile secretion and retention of hydrophobic and cytotoxic bile acids promote oxidative stress, hepatocellular damage, and ongoing inflammation, ultimately leading to bile duct destruction and fibrosis [65, 66]. Clinically, serum TBA levels are not only a hallmark of cholestasis but also a dynamic biomarker of disease severity and therapeutic response [67, 68]. Therefore, the observed reduction in TBA levels following siTIMP1‐LNP treatment suggests a dual benefit: attenuation of fibrogenic processes and broader improvement in hepatobiliary function, reinforcing the therapeutic potential of siTIMP1‐LNPs in chronic cholestatic diseases. Total bilirubin (TB) and cholesterol concentrations displayed favorable trends in the treated cohorts (Figure S8d), whereas alkaline phosphatase (ALP) levels remained stationary, suggesting that the therapeutic benefit on cholestasis may be selective to bile acid metabolism.

**FIGURE 7:**
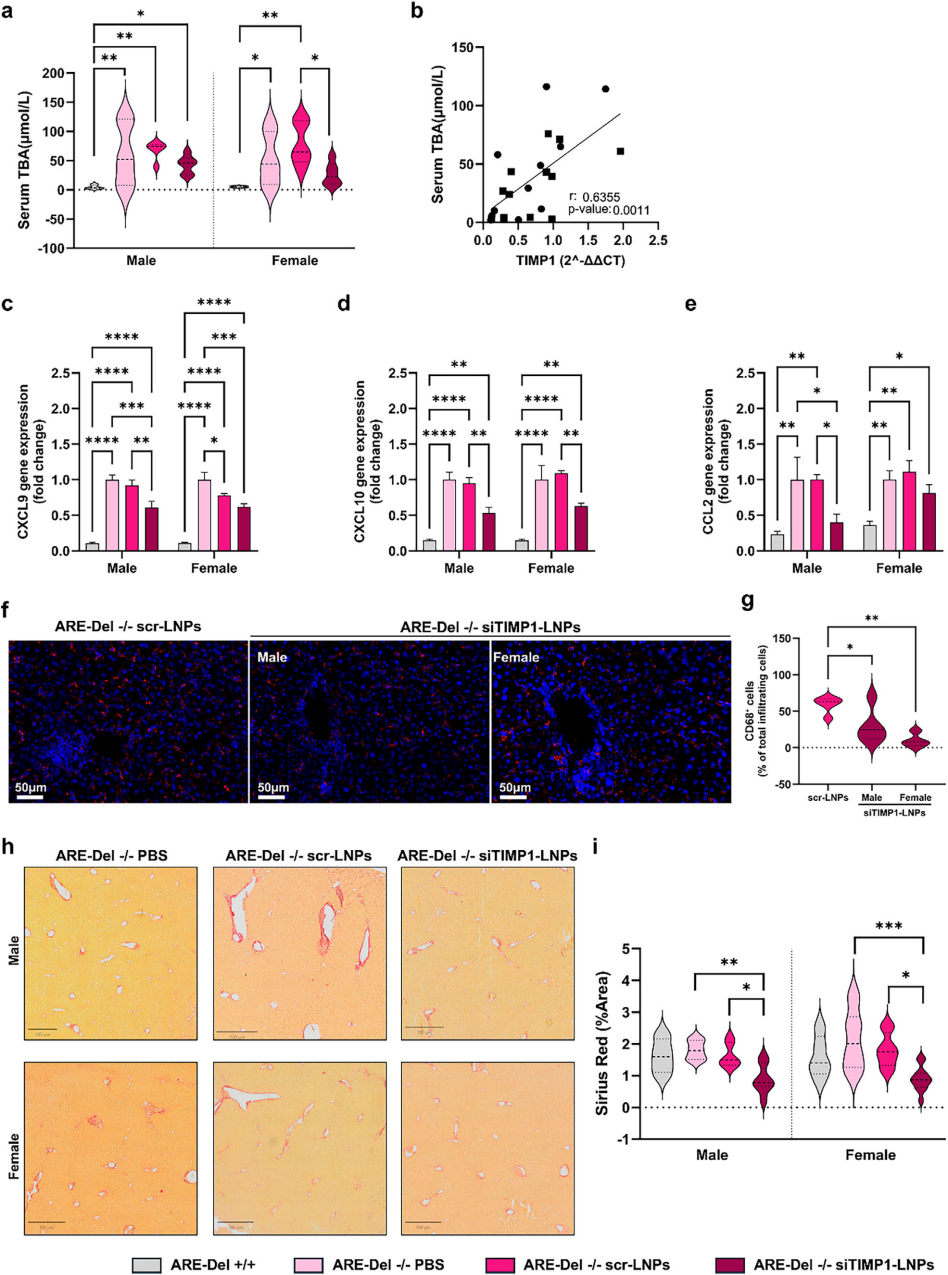
siTIMP1‐LNP treatment alleviates cholestasis, reduces macrophage infiltration, and mitigates liver fibrosis in ARE‐Del‐/‐ mice. (a) Quantification of serum TBA across experimental groups, shows a significant reduction in siTIMP1‐LNP‐treated mice, suggesting improved bile acid handling. (b) Positive correlation between hepatic Timp1 expression and serum TBA levels (r = 0.6355, p = 0.0011) supports the involvement of TIMP1 in cholestatic injury. (c–e) Quantitative RT‐PCR analysis of key hepatic chemokines Cxcl9, Cxcl10, and Ccl2 in whole liver tissue, which are downregulated following treatment, indicating reduced chemokine‐mediated recruitment of inflammatory monocytes and T cells. Data are presented as mean ± SEM (*n* = 3 animals per group) and analyzed by two‐way ANOVA followed by Šídák's multiple comparisons test. (f) Representative immunofluorescence images of CD68^+^ macrophages (red) and nuclei (Hoechst, blue) showing decreased infiltration in the siTIMP1‐LNP group, especially in females. Scale bar = 50 µm. (g) Whole‐slide morphometric quantification confirming a significant reduction in CD68^+^ cells relative to total infiltrating nuclei. (h) Representative Sirius Red staining of liver sections reveals reduced collagen deposition in siTIMP1‐LNP‐treated animals compared to PBS or scrambled control (scr‐LNPs). Scale bar = 500 µm. (i) Quantitative scoring of fibrosis confirms a significant reduction in fibrotic burden upon TIMP1 silencing in both sexes. Significant differences are defined as ^*^ *p* < 0.05, ^**^ *p* < 0.01, ^***^ *p* < 0.001, and ^****^ *p* < 0.0001.

These findings are consistent with the biochemical and histological improvements observed in treated mice, further emphasizing a sex‐biased therapeutic advantage. At the molecular level, TIMP1 silencing induced a reconfiguration of the hepatic immune microenvironment. The hepatic macrophage compartment, composed of resident KCs and monocyte‐derived macrophages (MoMs), responds dynamically to chemotactic signals, particularly those mediated by Cxcl9, Cxcl10, and Ccl2. These key chemokines were significantly downregulated in livers following TIMP1 silencing (Figure 7c–e), suggesting impaired recruitment of pro‐inflammatory monocytes and effector T cells. In particular, the reduction in Ccl2 implicates a blunted activation of the CCL2/CCR2 axis, a well‐established mediator of fibrogenic monocyte trafficking in cholestatic liver diseases. Indeed, in PBC, senescent cholangiocytes exhibit elevated CCL2 expression, facilitating the infiltration of CCR2^+^ inflammatory monocytes, which in turn exacerbate immune injury and activate HSCs toward a profibrotic phenotype [69, 70]. Although targeting CCR2 has shown efficacy in preclinical models, clinical translation has proven complex due to the pleiotropic and context‐dependent roles of this pathway in both fibrosis progression and resolution [71].

Immunofluorescence analysis confirmed these molecular findings: CD68^+^ macrophage infiltration in periportal and lobular regions was markedly reduced in treated mice, particularly in females (Figure 7f,g). Although hematoxylin and eosin (H&E) staining did not reveal clear improvements in lobular architecture following TIMP1 knockdown (data not shown), Sirius Red staining demonstrated a significant reduction in collagen deposition in siTIMP1‐LNP‐treated mice compared with controls (Figure 7h,i). These findings align with the current models of liver repair, which suggest that fibrosis regression could precede full architectural remodeling, as documented in both preclinical and clinical models of chronic liver disease [72].

The observed reduction in matrix content, in the absence of architectural normalization, suggests that TIMP1 silencing primarily facilitates early phases of fibrosis resolution, such as extracellular matrix degradation and modulation of the immune microenvironment, rather than triggering full parenchymal reorganization. This is consistent with the known kinetics of liver repair, where myofibroblast inactivation, matrix clearance, and immune reprogramming precede complete tissue remodeling [72].

Taken together, these data support a model in which TIMP1 silencing via targeted LNP not only restores ECM homeostasis by reactivating macrophage‐derived metalloproteinases but also limits bile acid accumulation and dampens chemokine‐mediated immune cell recruitment, contributing to a more resolutive immune environment. These multifaceted effects converge to attenuate fibrogenic remodeling in a cholestatic setting, reinforcing the therapeutic potential for targeting TIMP1 in macrophage‐driven liver pathologies]

## 3 Conclusion

Our study shows that modest changes in lipid composition can generate markedly different protein corona profiles, despite broadly comparable physicochemical properties of the underlying LNPs. LNP1 and LNP2 acquired coronas characterized by coordinated differences in apolipoproteins, complement, coagulation proteins, immunoglobulins, albumin, and vitronectin, and these formulations displayed the strongest hepatic accumulation. Rather than supporting a single‐protein mechanism, these findings indicate that liver tropism emerges from the collective biological identity encoded by the corona. Consistent with this interpretation, coronated LNP1 showed enhanced binding to MARCO, a scavenger receptor expressed by KC, and siTIMP1‐loaded LNP1 was efficiently taken up by this cell population, resulting in target silencing and downstream therapeutic effects in vivo. Together, these data support protein corona engineering as an alternative to conventional ligand or antibody conjugation for directing LNPs toward disease‐relevant hepatic cell populations. Notably, TIMP1 silencing also shifted the hepatic immune microenvironment toward a resolving phenotype, reducing key chemokines (Ccl2, Cxcl9, Cxcl10), increasing the expression of Il‐10 and Arg1, and alleviating bile acid accumulation. This dual effect on extracellular matrix homeostasis and immune regulation resulted in a significant reduction in liver fibrosis and macrophage infiltration. Figure 8 schematically illustrates the main steps from LNP synthesis to evaluation of their efficacy in the PBC model. This figure highlights the simplicity and translational potential of our strategy, which exploits spontaneous interactions with PC rather than requiring surface functionalization.

**FIGURE 8:**
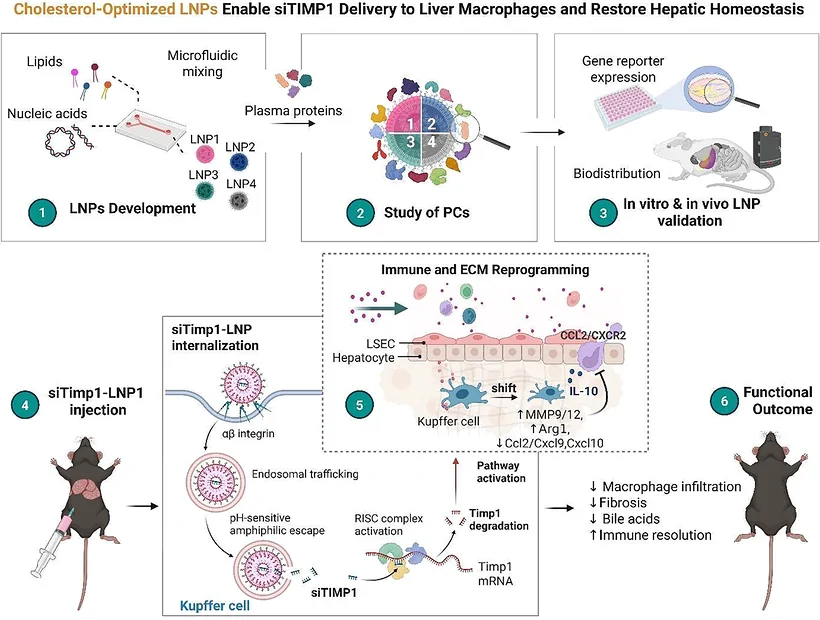
Schematic representation of the production and therapeutic mechanism of siTIMP1‐LNPs. (1) Lipids and nucleic acids (i.e., pDNA and siRNA) were mixed via microfluidics to generate four LNP formulations with varying lipid composition. (2) The LNPs were incubated with plasma proteins, and the resulting PC was characterized by nanoLC‐MS/MS. (3) Both coronated and bare LNPs were evaluated for TE and cytotoxicity, and bare LNPs were administered intraperitoneally (i.p.) in healthy mice to study in vivo biodistribution before the treatment of ARE‐Del‐/‐ with the best‐performing LNP loaded with siTIMP1. Upon i.p. injection (4), siTIMP1‐loaded LNPs are internalized by liver‐resident Kupffer cells, undergo endosomal trafficking, and escape the endosome through pH‐sensitive destabilization, enabling siRNA release and RISC complex activation. The resulting degradation of Timp1 mRNA reprograms Kupffer cells toward a resolving phenotype, characterized by increased expression of Mmp9, Mmp12, Il‐10, and Arg1, and downregulation of fibrogenic chemokines such as Ccl2, Cxcl9, and Cxcl10 (5). These molecular events converge in functional improvements (6), including reduced macrophage infiltration, decreased fibrosis and bile acid accumulation, and enhanced immune resolution. Figure Created in BioRender.

## 4 Experimental Section

### 4.1 LNPs Preparation

1,2‐dioleoyl‐3‐trimethylammonium‐propane (DOTAP), 3ß‐[N‐(N″,N″‐dimethylaminoethane)‐carbamoyl]cholesterol hydrochloride (DC‐Cholesterol), 1,2‐distearoyl‐sn‐glycero‐3‐phosphocholine (DSPC), 1,2‐dioleoyl‐sn‐glycero‐3‐phosphoethanolamine‐N‐polysarcosine‐25 (ammonium salt) (DOPE), 1,2‐dioleoyl‐sn‐glycero‐3‐phosphoethanolamine‐N‐[amino(polyethylene glycol)‐2000] (ammonium salt) (DOPE‐PEG 2000) and 1,2‐distearoyl‐sn‐glycero‐3‐phosphoethanolamine‐N‐[carboxy(polyethylene glycol)‐2000] (sodium salt) (DSPE‐PEG 2000) were purchased by Avanti research (Birmingham, AL, USA). Cholesterol was obtained from Sigma‐Aldrich (Merck, Darmstadt, Germany). LNPs were formulated by individually weighing each lipid component and dissolving them in ethanol to reach a final concentration of 12.5 mm. The lipids were then combined according to the molar ratios specified in Table 1. pDNA or siRNA was diluted using 25 mm citrate buffer at pH 4, at a concentration of 233 µg/mL. The aqueous nucleic acid solution was subsequently mixed with the ethanolic lipid phase through microfluidic mixing using the NanoAssemblr Ignite platform (Precision Nanosystems, Vancouver, Canada), applying a flow rate ratio of 1:3 (ethanol:aqueous) and a total flow rate of 5 mL min^−1^. To lower the ethanol content to approximately 25% v/v, the resulting LNP dispersion was dialyzed against 1× phosphate‐buffered saline (PBS) at 4°C for 19 h.

### 4.2 Physicochemical Characterization

After a 100‐fold dilution in ultrapure water, the hydrodynamic diameter and polydispersity index (PdI), and particle concentration of each formulation were measured by dynamic light scattering (DLS) using a Zetasizer Ultra Red (Malvern Panalytical). Zeta potential was determined by electrophoretic light scattering (ELS) using the same instrument.

### 4.3 Encapsulation Efficiency

RiboGreen RNA assay kit (Thermo Fisher Scientific, Waltham, MA, USA). Working solutions were prepared according to the manufacturer's instructions. LNP samples were diluted 1:150 in TE buffer or TE buffer containing Triton X‐100 (2% v/v) and loaded into a white bottomed 96‐well plate (Corning solid polystyrene, Sigma–Aldrich, St. Louis, MO, USA). Each sample was mixed in a 1:1 (v/v) ratio with the RiboGreen reagent. Following a 5‐min incubation at room temperature, fluorescence was measured using a Glomax Discovery System (Promega, Madison, WI, USA) with an excitation wavelength of 475 nm and emission between 500–550 nm. Encapsulation efficiency was calculated by comparing total RNA levels with the free siRNA content (measured in the absence of detergent). To quantify the total RNA content (encapsulated + unencapsulated) a calibration curve was performed using siRNA.

### 4.4 Shotgun Proteomics Sample Preparation

Protein pellets were processed following a previously employed protocol [73]. In brief, pellets were resuspended in 100 µL of 8 mol L^−1^ urea prepared in 50 mmol L^−1^ tris(hydroxymethyl)aminomethane hydrochloride (tris‐HCl) buffer, pH 7.8. Once fully solubilized, proteins were reduced with 2 µL of 200 mmol L^−1^ dithiothreitol for 1 h at 37°C, then alkylated with 8 µL of 200 mmol L^−1^ 2‐iodoacetamide for 1 h at room temperature in the dark. The reaction was quenched by adding 8 µL of 200 mmol L^−1^ dithiothreitol. Subsequently, the urea concentration was diluted to 1 mol L^−1^ with 50 mmol L^−1^ tris‐HCl buffer (pH 7.8), and proteins were digested overnight with 2 µg of trypsin at 37°C. The enzymatic reaction was terminated by adjusting the solution to pH 2.5 with trifluoroacetic acid. Peptide mixtures were then purified using Bond Elut C18 EWP solid‐phase extraction cartridges, dried under vacuum, and reconstituted in 100 µL of 0.1% formic acid.

### 4.5 Shotgun Proteomics Analysis

Peptide mixtures were subjected to nanoHPLC–MS/MS following the previous procedure with slight adjustments [74]. Analyses were carried out on an Ultimate 3000 nanoHPLC system coupled to an Orbitrap Elite mass spectrometer (Thermo Fisher Scientific). For each run, 20 µL of sample was loaded and concentrated online using a 300 µm i.d. × 5 mm Acclaim PepMap 100 C18 µ‐precolumn (Thermo Scientific) equilibrated with 99:1 (v/v) water/acetonitrile containing 0.1% trifluoroacetic acid, at a flow of 10 µL min^−^^1^. Peptide separation was performed on an EASY‐Spray analytical column (15 cm × 75 µm i.d., PepMap C18, 2 µm particle size, 100 Å pore size; Thermo Fisher Scientific), operated at 35°C and a flow rate of 300 nL min^−^^1^. Chromatographic resolution was achieved using a 100‐min gradient with mobile phases A (water with 0.1% formic acid) and B (acetonitrile with 0.1% formic acid): 1% B for 5 min, ramped to 5% B over 2 min, then increased to 35% B across 90 min. The column was subsequently flushed with 90% B for 10 min and re‐equilibrated at 1% B for 30 min. The Orbitrap Elite was operated in data‐dependent acquisition mode. Full MS scans were recorded over 300–2000 m/z at a resolution of 30000 (FWHM at m/z 400). The ten most intense precursor ions per cycle were selected for higher‐energy collisional dissociation (HCD) at 35% normalized collision energy, using a 2 m/z isolation window, with spectra acquired at 15000 resolution. Singly charged and unassigned ions were excluded, and dynamic exclusion was enabled (repeat count = 1, repeat duration = 30 s, exclusion duration = 70 s). Each sample was analyzed in triplicate. Raw MS/MS data acquired with Xcalibur software (version 2.2 SP1.48, Thermo Fisher Scientific) were processed in MaxQuant (v1.6.3.4) against the Swiss‐Prot human protein database [75]. Database search parameters included: enzyme = trypsin, cleavage at Lys/Arg, up to 2 missed cleavages; fixed modification = carbamidomethylation (Cys); variable modifications = oxidation (Met), acetylation (protein N‐terminus), and deamidation (Asn, Gln). Mass tolerance was set to 10 ppm for precursors and 20 ppm for fragments. Protein identification required at least one razor/unique peptide. The false discovery rate was controlled at 1% for peptide‐spectrum matches, peptides, and proteins. Quantitative analysis was performed using the MaxQuant output file ′proteinGroups.

### 4.6 In Vitro Validation of LNPs

HEK‐293 cells (RRID:CVCL_0045; ATCC, Cat. No. CRL‐1573) were purchased from the American Type Culture Collection (ATCC) (Manassas, VA, USA) and cultured in DMEM supplemented with 10% fetal bovine serum (FBS) and 1% penicillin–streptomycin. Although the exact date of purchase is unavailable, the cell line has been stably maintained in our laboratory and tested negative for Mycoplasma contamination. For TE and cell viability experiments, cells were seeded in 96‐well plates at a density of 1 × 10^5^ cells/well and incubated overnight at 37 °C with 5% CO_2_. The treatment was performed in 96 multiwell plates treated with 0.2 µg/well of formulated pDNA (pmirGlo) coding for firefly luciferase delivered by LNPs and their human plasma coronated counterpart. The culture media was replaced with calcium‐free, serum‐free DMEM containing the LNPs, and the cells were incubated at 37 °C with 5% CO_2_. After 3 h, the treatment was completely replaced with standard DMEM supplemented with 10% FBS and 1% Penicillin‐Streptomycin. After 48 h, cell viability was assessed using the CellTiter‐Fluor Cell Viability Assay (Promega) according to the manufacturer's instructions. The cells were lysed using 20 µL Passive lysis buffer (Promega) each well, 10 µL was used for Pierce BCA Protein Assay (Thermo Scientific), and the remaining volume was used for Luciferase Assay System (Promega). The luminescence was quantified with the GloMax Discover Microplate Reader (Promega).

### 4.7 In Vitro Validation of Gene Silencing

Murine macrophages, RAW 264.7 cells (RRID:CVCL 0493; ATCC, Cat. No. TIB‐71) were purchased from the ATCC (Manassas, VA, USA) and cultured in DMEM supplemented with 10% FBS and 1% P/S at 37°C in a humidified 5% CO_2_ incubator. The line has been stably propagated in our laboratory and regularly screened to ensure the absence of Mycoplasma contamination. RAW 264.7 cells were used for in vitro validation of LNP‐mediated siRNA delivery and macrophage activation studies, representing a widely accepted model for murine macrophages. For treatment, cells were seeded in 24‐well plates at a density of 5 × 10^5^ cells/well and incubated overnight. For silencing, cells were treated with 100 ng/ml LPS for 2 h and then with 0.125, 0.25, and 0.5 nmol of siRNA‐Timp1‐LNP in complete DMEM. After 24 h, total RNA was extracted using TRIzol (Thermo Fisher).

Primary KCs were isolated from 24‐week‐old ARE‐Del‐/‐ female mice (as described in the Isolation of Liver NPCs and KCs section). After adherence‐based purification, KCs were seeded at 2.5 × 10^5^ cells/well in 24‐well plates and cultured for 24 h in DMEM containing 10% FBS, 1% P/S, and 50 ng/mL recombinant M‐CSF (PeproTech). For silencing, cells were treated with 0.125, 0.25, and 0.5 nmol of siRNA‐Timp1‐LNP in complete DMEM. After 24 h, total RNA was extracted using TRIzol (Thermo Fisher).

### 4.8 Quartz Crystal Microbalance Experiment

The interaction between coronated LNP1 and MARCO was investigated by Quartz Crystal Microbalance (QCM) using an openQCM Q‐1 instrument (openQCM, Italy). Measurements were carried out with 10 MHz AT‐cut quartz crystal sensors (AT10‐14‐12‐AU‐WRAP, Novaetech S.r.l., Italy), featuring a 14 mm quartz disk coated with an 11.5 mm gold/titanium (Au/Ti) electrode. Before functionalization, the crystals were cleaned by immersion in acetone, followed by rinsing with 70% ethanol and distilled water (d‐H_2_O). MARCO immobilization was achieved by incubating the sensors overnight at room temperature in acetate buffer containing the receptor at a concentration of 0.005 mg/mL. After incubation, excess protein was removed by rinsing the crystals with distilled water. The functionalized sensors were mounted in a microfluidic flow cell connected to Tygon tubing and a syringe pump, allowing precise control of solution delivery. Resonance frequency changes were continuously recorded using the manufacturer's acquisition software. Prior to sample injection, the system was equilibrated by flowing 0.5 mL of distilled water through the chamber at 25 µL min^−1^ until a stable baseline was obtained. Coronated LNP1 suspensions (0.005 mg/mL) were then introduced into the flow cell (1 mL) at a flow rate of 10 µL min^−1^. After the binding phase, the sensor surface was washed with 0.5 mL of distilled water at 25 µL min^−1^ to remove loosely associated material. As negative controls, bare LNP1 suspensions (0.005 mg/mL) were injected onto MARCO‐functionalized sensors under identical experimental conditions, followed by the same washing procedure. Changes in adsorbed mass (Δm) were calculated from the measured resonance frequency variation (Δf) according to the Sauerbrey equation [76]:

(1) $$\Delta m=-\frac{A\sqrt{\rho_{q}\mu_{q}}}{2f_{0}^{2}}\Delta f$$

where *f*_0_ is the fundamental frequency, *A* is the active area, ρ_q_ is the quartz density (2.65 g/cm^3^ at 25°C), and μ_q_ is the shear modulus (2.95 × 10^11^ g/cm·s^2^).

### 4.9 Animal Model and Ethics

All animal procedures were conducted in compliance with institutional guidelines and approved by the Mario Negri Institute Animal Care and Use Committee (IACUC) and the Italian National Institute of Health (code no. 49/2021‐PR and 1057/2023 PR). All procedures were conducted in accordance with Italian Governing Law (D.lgs 26/2014) and the EEC Council Directive 2010/63/UE. Mice were purchased from Charles River and acclimatized for at least a week before investigation. Mice were housed under specific pathogen‐free (SPF) conditions in the Institute's animal care facilities, with a controlled temperature (21 ± 1°C) and humidity (55 ± 10%), with ad libitum access to food and water, and a 12‐h light/dark cycle. A veterinarian regularly checked their welfare and reviewed experimental protocols.

### 4.10 In Vivo Safety and Biodistribution Studies

To evaluate systemic and organ‐specific biodistribution, twenty‐four‐week‐old CD1 mice (Charles River, Italy; n = 4 per group) received a single intraperitoneal (i.p.) injection of fluorescently labeled LNPs (1.23 mg kg^−1^, equivalent to a 5 µg dose of encapsulated mRNA). Control mice were administered an equivalent volume of PBS. At defined time points (30 min, 2, and 4 h post‐injection), animals were euthanized with an overdose of ketamine (150 mg kg^−1^) and medetomidine (2 mg kg^−1^). Blood was immediately collected into EDTA tubes and centrifuged to isolate plasma, while major organs (liver, spleen, kidney, lung, and brain) were collected. All samples were subsequently processed for *ex vivo* imaging or snap‐frozen in dry ice for further analysis.

For *ex vivo* imaging, harvested solid organs and aliquots of isolated plasma (50 µL transferred into black 96‐well microplates) were imaged using the IVIS Lumina III system (PerkinElmer) with the following settings: excitation filter range 640–680 nm, emission filter range 695–770 nm (optimized for Texas Red detection), exposure time 2 s, binning factor 4, and f/Stop 2. Spectral unmixing, image normalization, and signal quantification, expressed as average radiant efficiency (p/s/cm^2^/sr)/(µW/cm^2^), were carried out using Living Image software (version 4.3.1).

To provide an orthogonal and highly sensitive assessment of systemic circulation kinetics, plasma samples were further characterized via spectrofluorometric profiling using a microplate reader (PerkinElmer). Fluorescence emission spectra were acquired across a wavelength range of 585 to 650 nm under a fixed excitation wavelength. Quantitative evaluation of circulating formulations was performed by measuring the absolute fluorescence intensity at the peak emission wavelength (610 nm), subtracting the auto‐fluorescence background obtained from untreated control plasma (CTR) to eliminate matrix effects.

### 4.11 RNA‐Seq Data Analysis and Gene Enrichment

To identify global drivers of liver fibrosis and macrophage‐specific dysfunction, public RNA‐sequencing datasets were retrieved from the Gene Expression Omnibus (GEO) repository. The dataset GSE76309 included liver samples from wild‐type (WT) and ARE‐Del‐/‐ (HO) male and female mice at 24 weeks of age were analyzed alongside bulk RNA‐seq data from KCs isolated from CCl4‐induced fibrotic livers (GSE226946). Differentially expressed genes (DEGs) were identified using the limma package in RStudio, applying a cut‐off of |log2FC| > 1 and adjusted p‐value < 0.1 for dataset cross‐comparison. Volcano plots were generated to visualize DEGs in HO vs. WT in both sexes. Hierarchical clustering and heatmaps were generated using pheatmap, with unsupervised clustering of samples and genes based on Euclidean distance.

To explore how global liver transcriptional changes relate to macrophage‐specific responses, the whole‐liver DEGs were intersected with the fibrotic KC dataset. The resulting core genes were stratified into distinct categories based on their modulation profile (sex‐specific, sex‐independent, unchanged, or low expressed in KCs) and ranked using a custom Balanced Relevance Score. This score integrated network connectivity (number of associated Gene Ontology terms) and biological relevance weighting factors targeting core pathological mechanisms (e.g., extracellular matrix remodeling and inflammation). Functional annotation and pathway over‐representation analyses of the prioritized sex‐independent upregulated gene set were performed using Metascape (https://metascape.org), considering terms with an adjusted p‐value < 0.05 as statistically significant.

### 4.12 Chronic Treatment

ARE‐Del‐/‐ and WT mice of both sexes were randomly assigned to different experimental groups (21 weeks of age, n = 3 per group). Animals were weighed and subjected to baseline blood collection three days prior to treatment initiation (Day ‐3). The therapeutic regimen began at Day 0 and consisted of 12 intraperitoneal (i.p.) injections of LNPs (1.23 mg kg^−1^) administered at days 0, 3, 5, 8, 10, 12, 15, 17, 19, 22, 24, and 26, spanning a 5‐week experimental window (Figure 4e). Animal body weight was monitored throughout the study to evaluate tolerability. On Day 29 (three days after the final dose), mice were euthanized, and blood, spleen, and liver were collected for downstream analyses. A portion of each liver was fixed in 10% neutral buffered formalin for 24 h and embedded in paraffin for histological evaluation, another was snap‐frozen for molecular and biochemical assays, and a defined lobe was processed fresh for the isolation of hepatic and non‐parenchymal cells, including KCs, to assess immune cell phenotype and cellular nanoparticle uptake.

### 4.13 Isolation of Liver NPCs and KCs

After completion of chronic LNP treatment, hepatic cells were isolated from controls or LNP‐treated ARE‐Del‐/‐ mice. Following euthanasia, livers were perfused via the inferior vena cava with 10 mL of sterile PBS to remove residual blood. The organs were minced into small pieces and enzymatically digested at 37°C for 15 min in 10 mL of RPMI GlutaMAX medium (Gibco) containing 200 U/mL of collagenase type IV and 100 U/mL of DNAse I (Sigma‐Aldrich). The digested fragments were passed through an 18G needle using a syringe and filtered through a 70 µm cell strainer to obtain a single‐cell suspension. The suspension was centrifuged at 50 × g for 3 min at 4°C to pellet the parenchymal hepatocyte fraction (HCs). The supernatant, enriched in non‐parenchymal cells (NPCs), was collected and centrifuged at 400 × g for 6 min at 4°C. For hepatocytes and the NPC fraction were immediately lysed in TRIzol Reagent (Thermo Fisher) and stored for RNA extraction. For KCs isolation, another portion of NPCs was seeded in 6‐well plate at a density of 1–3×10^7^ cells/well in complete DMEM and incubated at 37°C with 5% CO_2_ for 2 h. Non‐adherent cells were gently removed by washing with PBS. The adherent cells, enriched in KCs, were maintained in complete DMEM supplemented with 50 ng/mL recombinant mouse M‐CSF and used for downstream assays.

### 4.14 Gene Expression and Correlation Analysis by qPCR

To evaluate TIMP1 silencing effects and identify co‐regulated genes, we performed qPCR on total RNA from livers or isolated cell types of WT and ARE‐Del‐/‐ mice (both sexes, treated with PBS, scrambled siRNA, or siTIMP1‐LNPs). RNA was extracted with TRIzol and reverse‐transcribed (1 µg) using the High‐Capacity cDNA kit (Applied Biosystems). qPCR was performed on QuantStudio 5 using two approaches:

TaqMan Array Cards (Custom 96‐well): included fibrosis‐, ECM‐, immune‐, and apoptosis‐related genes, plus housekeeping controls (Hprt, Gapdh, 18S, Gusb; full list in Table S4). Data were analyzed by ΔΔCt method, with Hprt as the reference gene after assessment of housekeeping stability. SYBR Green Assays: used to quantify additional targets not included in the array and for gene expression profiling in isolated liver cell subsets (listed in Table S5). Primer specificity and efficiency were validated; melt curve analysis confirmed single‐product amplification. For correlation analysis, ΔCt values were used to generate Pearson correlation matrices between Timp1 and other genes. Hierarchical clustering and heatmaps were generated in R‐studio (pheatmap package) to identify co‐regulated gene clusters.

### 4.15 Histology and Immunofluorescence

Sirius Red staining was used to evaluate the distribution of collagen fibers in liver tissue sections from mice derived from chronic multiple treatment. Liver tissues were fixed in 10% neutral buffered formalin for 24 h, embedded in paraffin, and sectioned at 4–5 µm thickness. Sections were incubated in Picro‐Sirius Red solution (Bio‐Optica) for 60 min, rinsed with acidified water to remove excess dye, dehydrated through graded ethanol, cleared in xylene, and mounted with a xylene‐based medium (DPX, Sigma‐Aldrich). Digital acquisition of entire liver sections was performed using a Virtual Slide Microscope (Olympus). Collagen‐positive areas were quantified using QuPath software (version 0.5.1), applying threshold‐based segmentation and blinded analysis. Results were expressed as a percentage of collagen area over total tissue area.

Immunofluorescence analysis to label macrophages was performed on cryosections (10 µm) from frozen liver tissues obtained both in the single‐dose treatment study to understand the colocalization with LNP1, and in the multiple‐dose treatment study to quantify the amount of infiltrating cells. Samples from multiple‐dose treatment were then incubated for 1 h in blocking solution (PBS containing 10% normal goat serum [NGS]and 0.1% Triton X‐100), followed by overnight incubation at 4°C with a primary anti‐CD68 antibody (1:200, Serotec) diluted in PBS containing 3% NGS and 0.1% Triton X‐100. Samples from the single‐dose treatment group were subjected to the same experimental procedure, except that Triton X‐100 was omitted during the blocking and primary antibody incubation steps. This modification was necessary because the detergent disrupts lipid membranes and would therefore also compromise the integrity of the LNPs. The next day, sections were washed and incubated with a goat anti‐rat secondary antibody (1:500, Invitrogen) conjugated with Alexa Fluor 488 (for single treatment) or with Alexa Fluor 546 (for multiple treatments) for 1 h at room temperature in PBS + 1% NGS. Nuclei were counterstained with Hoechst 33 258 (2 µg/mL in PBS for 10 min). Images were acquired using a Virtual Slide Microscope (Olympus), equipped with excitation lasers at 405 (Hoechst), 48, and 546 nm. Quantification of CD68^+^ area in tissues derived from the multiple‐dose treatment study was performed on virtual slide scans using QuPath.

### 4.16 Serum Biochemical Analysis

Blood samples were collected from mice at sacrifice (3 days post‐final injection in the chronic treatment protocol) and transferred into serum separator tubes. Samples were left to clot at room temperature for 20–30 min and subsequently centrifuged at 2000 × g for 10 min at 4°C. The resulting serum was carefully transferred into clean tubes and stored at −80 °C until analysis.

Serum biochemical parameters, including alkaline phosphatase (ALP), total bilirubin (TB), total bile acids (TBA), total cholesterol (CHOL), aspartate aminotransferase (AST), alanine aminotransferase (ALT), total protein (TP), albumin (ALB), globulin (GLOB), and albumin/globulin (A/G) ratio, were measured using a fully automated clinical chemistry analyzer (Element RC, Scil Animal Care Company S.r.l) according to the manufacturer's instructions.

The AST/ALT ratio was calculated manually for each animal based on the measured enzyme activities. Data were analyzed separately for male and female mice to account for sex‐specific differences in response to treatment.

## Author Contributions

N.G.S. and S.R. contributed equally. N.G.S., D.P., A.L., P.B., and G.C. conceived the research; N.G.S., S.R., M.B.V., L.A., A.C., E.Q., A.M., and L.D. performed experiments and collected data; C.A.S., P.I., and G.C. acquired funding; N.G.S., S.R., L.D., A.L.C., M.P., P.B., and G.C. analyzed results; N.G.S., S.R., P.B., and G.C. wrote the manuscript with input from all authors. All authors approved the final version.

## Funding

NextGenerationEU, through the Italian Ministry of University and Research, under the PNRR – M4C2‐I1.3 Project PE_00000019 ‘HEAL ITALIA’ (Giulio Caracciolo, CUP: B53C22004000006; Carlo Scirè, CUP: H43C22000830006). The AIRC Foundation for Cancer Research in Italy is acknowledged for supporting G.C. (Project IG2025 – ID 32500) and S.R. through an AIRC Fellowship for Italy Post‐Doc grant (Project ID 32938‐2025).

## Conflicts of Interest

The authors declare no conflicts of interest.

## Supporting information




**Supporting File**: smll75537‐sup‐0001‐SuppMat.docx.

## Data Availability

The data that support the findings of this study are available from the corresponding author upon reasonable request.
