# Supplementary material for: Protein Corona Engineering of Lipid Nanoparticles Enables Efficient Functional Gene Silencing in the Liver
Source: Small. 2026 Sep 15;22(62):e75537. doi: 10.1002/smll.75537 (PMC13742893; doi:10.1002/smll.75537)
Supplement: Supplementary file 1 [file SMLL-22-e75537-s001.docx]

S **Protein Corona Engineering of Lipid Nanoparticles Enables Efficient Functional Gene Silencing in the Liver**

*Naths G. Sukubo ^1^, Serena Renzi ^2^, Martina B. Violatto ^3^, Leila Altigieri^3^, Anna L. Capriotti ^4,5^, Andrea Cerrato ^4,5^, Aldo Laganà ^4,5^, Luca Digiacomo ^6^, Erica Quagliarini ^2^, Antonio Minopoli^7^, Massimiliano Papi ^7,8^, Daniela Pozzi ^2,9, *^, Carlo A. Scirè^1^, Pietro Invernizzi ^1, 8^, Paolo Bigini ^3,*^, Giulio Caracciolo ^2, 9 *^.*

E-mail: [daniela.pozzi@uniroma1.it](mailto:daniela.pozzi@uniroma1.it), [paolo.bigini@marionegri.it](mailto:paolo.bigini@marionegri.it), [giulio.caracciolo@uniroma1.it](mailto:giulio.caracciolo@uniroma1.it),

N. G. Sukubo, C. A. Scirè, P. Invernizzi

School of Medicine and Surgery, University of Milano-Bicocca, Via Cadore, 48, 20900 Monza, Italy.

S. Renzi, Erica Quagliarini, D. Pozzi, G. Caracciolo

Department of Molecular Medicine, Sapienza University of Rome, Viale Regina Elena 291, 00161 Rome, Italy

M. B. Violatto, L. Altigieri, P. Bigini

Department of Biochemistry and Molecular Pharmacology, Istituto di Ricerche Farmacologiche “Mario Negri” IRCCS, Via Mario Negri, 2, 20156 Milano, Italy
A. L. Capriotti, A. Cerrato, A. Laganà

Department of Chemistry, Sapienza University of Rome, Piazzale Aldo Moro 5, 00185, Rome, Italy

A. L. Capriotti, A. Cerrato, A. Laganà

Interuniversity Consortium INBB – Biostructures and Biosystems National Institute, Via dei Carpegna 19, 00165, Rome, Italy

L. Digiacomo

Department of Medical Surgical Sciences and Biotechnologies, Sapienza University of Rome, Corso della Repubblica 79, Latina, 04100, Italy

Antonio Minopoli, Massimiliano Papi

Department of Neuroscience, Catholic University of Sacred Heart, Largo Francesco Vito 1, 00168 Rome, Italy.

P. Invernizzi

Division of Gastroenterology, Center for Autoimmune Liver Diseases, European Reference Network on Hepatological Diseases (ERN RARE-LIVER), IRCCS Fondazione San Gerardo Dei Tintori, Monza, Italy

Massimiliano Papi

Fondazione Policlinico Universitario A. Gemelli, IRCCS, Rome 00168, Italy.

D. Pozzi, G. Caracciolo

Center for Nanotechnology Applied to Engineering (CNIS), Sapienza University of Rome, Rome 00185, Italy

**Funding**: NextGenerationEU, through the Italian Ministry of University and Research, under the PNRR - M4C2-I1.3 Project PE_00000019 'HEAL ITALIA' (Giulio Caracciolo, CUP: B53C22004000006; Carlo Scirè, CUP: H43C22000830006).

**Keywords**: Lipid Nanoparticles, Protein Corona, Liver-Targeted Delivery, TIMP1 Gene Silencing, Primary Biliary Cholangitis, Microfluidic Mixing

.

**Figure S1. Protein Corona Composition across LNP formulations**. The protein content of LNP-associated PCs was evaluated using nano-liquid chromatography tandem mass spectrometry and reported as relative protein abundance (RPA%). Identified proteins were classified into functional categories, while the most abundant individual proteins, such as Albumin and Vitronectin, were highlighted separately using distinct colors. Representation of the RPA% of each functional category of protein composing the PC of the studied LNPs. Total RPA%=100.

**Figure S2.** **Spectrofluorometric profiling of plasma circulation kinetics.** **(a–d)** Emission spectra (585-650 nm) of plasma samples collected at 30 minutes, 2, and 4 hours post-intraperitoneal injection from mice treated with: **(a)** LNP1, **(b)** LNP2, **(c)** LNP3, and **(d)** LNP4, compared to untreated controls (CTR). **(e)** Quantitative comparison of absolute plasma fluorescence intensity extracted at the peak emission wavelength (610 nm) across all formulations and kinetics time points. Data are expressed as mean +/- SEM (n=4 per group). Statistical significance was assessed using two-way ANOVA with Tukey’s multiple comparisons test (*p < 0.05, **p < 0.01, and ***p < 0.001 compared to the untreated control group, CTR).

**Figure S3**. **Quartz crystal microbalance analysis of pristine and coronated LNP1 binding to immobilized MARCO**. **(a)** Schematic representation of the QCM assay, in which MARCO was immobilized on the gold-coated sensor surface and subsequently exposed to pristine or coronated LNP1. **(b)** Representative binding kinetics showing the change in adsorbed mass (Δm) over time for coronated LNP1 (dashed pink curve) and pristine LNP1 (solid pink curve). Coronated LNP1 produced a markedly greater mass increase than its pristine counterpart, indicating that protein corona formation enhances the interaction of LNP1 with surface-immobilized MARCO.

**Figure S4. Intrahepatic distribution kinetics and macrophage spatial relationship of LNP1.** Representative *ex vivo* fluorescence microscopy images of liver sections from untreated mice (Ctr) and mice administered with Texas Red-labeled LNP1, harvested at 30 min, 2 h, and 4 h post-injection. The panel includes two-channel merges (cell nuclei + LNP1, left columns), full three-channel merges (cell nuclei + macrophages + LNP1, middle columns), and corresponding high-magnification areas (right columns, cropped from the dashed white boxes). The blue signal corresponds to cell nuclei (Hoechst 33258); red signal represents LNP1 formulations (Texas Red); green signal identifies the lysosomal compartment of resident macrophages/Kupffer cells (Alexa Fluor 488, anti-CD68 antibody). Scale bars: 50 μm (low magnification) and 20 μm (high magnification crops).

**Figure S5. Transcriptomic profiling of liver fibrosis in ARE-Del-/- mice.** **(a)** Heatmap of differentially expressed genes (DEGs) in liver tissue from female and male ARE-Del-/- (HO) mice versus wild-type (WT) controls (GSE76309). Hierarchical clustering reveals clear segregation between groups based on genotype and sex. **(b)** Volcano plots showing significantly upregulated (red) and downregulated (blue) genes in female and male HO vs. WT comparisons. A more pronounced transcriptional shift was observed in females. **(c)** Intersection of upregulated DEGs across sexes identifies common genes associated with fibrogenesis. **(d)** Gene ontology enrichment analysis (Metascape) of shared upregulated genes highlights pathways related to vasculature development, angiogenesis, MAPK signaling, and cell migration. Enrichment is expressed as –log10(p-value).

**Figure S6. Physicochemical Characterization of siRNA-LNPs.** **(a)** Size (Peak 1), **(b)** Polydispersity Index (PdI), **(c)** Z-potential and **(d)** Encapsulation Efficiency (EE%) of LNPs encapsulating scrambled (scr-LNPs) or specific TIMP1 siRNA (siTIMP1-LNPs) were studied after the preparation.

**Figure S7. TIMP1 co-regulated gene network in liver tissue.** Heatmap of pairwise Pearson gene expression correlation coefficients between Timp1 and a panel of fibrosis-, inflammation-, immune-, and ECM-related genes across experimental groups. Hierarchical clustering identified three distinct gene clusters (colored bars on the left) reflecting patterns of co-regulation. Red and blue indicate positive and negative correlations, respectively. The analysis was performed using ΔCt values; color intensity reflects the strength of the correlation (scale: −1 to +1). Clustering and visualization were performed in R using the pheatmap package.

**Figure S8.** ***In vivo* safety profile of a-Timp1-LNPs following chronic administration in ARE-Del-/- mice. (a)** Body weight change (%) over time in male and female mice during the 5-week experimental window. **(b)** Body weight viability, liver index, and spleen index at the end of treatment. **(c)** Serum protein panel including albumin, globulin, total protein, and albumin/globulin ratio. **(d)** Liver function and lipid-related serum parameters: alkaline phosphatase (ALP), total bilirubin (TB), and total cholesterol. **(e)** Hepatocellular injury markers (ALT, AST) and AST/ALT ratio. Data are shown separately for males and females. Bars represent mean ± SEM; each dot represents one mouse. Statistical analysis was performed using one-way ANOVA followed by Tukey’s post hoc test. *p<0.05, **p<0.01.

**Table S1.** List of proteins identified in the PC of LNPs. Proteins listed in alphabetical order. Those present at relative abundances below 0.001% across all LNP samples are not included

|  | LNP1 | LNP2 | LNP4 | LNP3 |
| --- | --- | --- | --- | --- |
| A1BG | 0.160% | 0.040% | 0.000% | 0.010% |
| A2M | 0.580% | 1.050% | 0.140% | 0.410% |
| ACAN | 0.000% | 0.000% | 0.000% | 0.010% |
| ACTA1; ACTC1; ACTA2; ACTG2 | 0.000% | 0.000% | 0.001% | 0.006% |
| AFM | 0.050% | 0.060% | 0.000% | 0.010% |
| AGT | 0.040% | 0.100% | 0.050% | 0.040% |
| AHSG | 0.000% | 0.460% | 0.150% | 0.230% |
| AHSG | 0.280% | 0.000% | 0.000% | 0.000% |
| Albumin | 45.300% | 24.300% | 16.800% | 22.400% |
| AMBP | 0.050% | 0.050% | 0.100% | 0.680% |
| APOA1 | 5.990% | 5.840% | 4.350% | 3.910% |
| APOA2 | 1.480% | 1.550% | 2.280% | 1.110% |
| APOA4 | 0.330% | 1.390% | 1.400% | 0.740% |
| APOB | 0.110% | 2.180% | 1.770% | 1.230% |
| APOC1 | 0.530% | 0.170% | 0.110% | 0.050% |
| APOC2 | 0.270% | 2.860% | 4.800% | 1.000% |
| APOC3 | 1.440% | 2.140% | 2.700% | 0.330% |
| APOC4 | 0.000% | 0.020% | 0.020% | 0.020% |
| APOD | 0.740% | 1.480% | 0.360% | 0.050% |
| APOE | 0.340% | 1.050% | 0.680% | 0.410% |
| APOF | 0.000% | 0.020% | 0.020% | 0.050% |
| APOH | 0.000% | 0.050% | 0.020% | 0.020% |
| APOL1 | 0.000% | 0.030% | 0.020% | 0.040% |
| APOM | 0.020% | 0.310% | 0.160% | 0.050% |
| ATRN | 0.000% | 0.000% | 0.000% | 0.000% |
| AZGP1 | 0.030% | 0.000% | 0.000% | 0.000% |
| C1QA | 0.000% | 0.010% | 0.009% | 0.000% |
| C1QB | 0.080% | 0.110% | 0.090% | 0.050% |
| C1QC | 0.070% | 0.100% | 0.120% | 0.060% |
| C1R | 0.010% | 0.060% | 0.030% | 0.020% |
| C1S | 0.020% | 0.050% | 0.040% | 0.050% |
| C2 | 0.000% | 0.000% | 0.000% | 0.000% |
| C3 | 0.810% | 12.350% | 17.440% | 10.130% |
| C4A | 0.000% | 0.012% | 0.015% | 0.008% |
| C4B | 0.190% | 2.200% | 2.470% | 1.490% |
| C4BPA | 0.180% | 0.290% | 0.260% | 0.690% |
| C4BPB | 0.000% | 0.000% | 0.000% | 0.021% |
| C5 | 0.000% | 0.190% | 0.320% | 0.110% |
| C6 | 0.020% | 0.070% | 0.050% | 0.060% |
| C7 | 0.010% | 0.000% | 0.008% | 0.006% |
| C8A | 0.010% | 0.020% | 0.020% | 0.030% |
| C8B | 0.000% | 0.003% | 0.011% | 0.022% |
| C8G | 0.040% | 0.020% | 0.020% | 0.030% |
| C9 | 0.020% | 0.210% | 0.260% | 0.140% |
| CD5L | 0.040% | 0.020% | 0.010% | 0.030% |
| CELSR2 | 0.000% | 0.000% | 0.000% | 0.000% |
| CFB | 0.100% | 0.070% | 0.020% | 0.060% |
| CFH | 0.170% | 1.340% | 1.050% | 1.180% |
| CFHR1 | 0.000% | 0.006% | 0.023% | 0.026% |
| CFI | 0.010% | 0.000% | 0.000% | 0.000% |
| CFP | 0.000% | 0.210% | 0.220% | 0.180% |
| CHMP4A | 0.000% | 0.040% | 0.030% | 0.000% |
| CLU | 0.040% | 0.530% | 0.440% | 0.980% |
| CNDP1 | 0.000% | 0.000% | 0.000% | 0.040% |
| COMP | 0.000% | 0.000% | 0.000% | 0.080% |
| CP | 0.080% | 0.860% | 0.710% | 0.460% |
| CRP | 0.120% | 0.270% | 0.200% | 0.030% |
| DCD | 0.000% | 0.036% | 0.062% | 0.056% |
| DNAJC13 | 0.000% | 0.000% | 0.000% | 0.030% |
| F10 | 0.000% | 0.003% | 0.007% | 0.007% |
| F12 | 0.020% | 0.000% | 0.000% | 0.000% |
| F13A1 | 0.000% | 0.001% | 0.000% | 0.031% |
| F2 | 0.117% | 0.339% | 0.607% | 1.137% |
| F9 | 0.000% | 0.001% | 0.010% | 0.185% |
| FCN3 | 0.010% | 0.000% | 0.000% | 0.000% |
| FGA | 1.641% | 4.959% | 3.996% | 4.117% |
| FGB | 1.080% | 2.640% | 2.046% | 2.234% |
| FGG | 1.523% | 2.813% | 2.251% | 2.283% |
| FLG | 0.000% | 0.002% | 0.004% | 0.000% |
| FLG2 | 0.000% | 0.005% | 0.003% | 0.006% |
| FN1 | 0.000% | 0.560% | 0.320% | 0.700% |
| GC | 0.400% | 0.080% | 0.020% | 0.060% |
| GSN | 0.020% | 0.186% | 0.262% | 0.523% |
| HABP2 | 0.000% | 0.030% | 2.700% | 4.020% |
| HBA1 | 0.130% | 0.040% | 0.050% | 0.080% |
| HBB | 0.220% | 0.080% | 0.210% | 0.160% |
| HP | 4.030% | 3.630% | 0.560% | 0.960% |
| HPR | 0.040% | 0.040% | 0.010% | 0.010% |
| HPX | 0.790% | 0.010% | 0.010% | 0.020% |
| HRG | 0.040% | 0.040% | 0.010% | 0.090% |
| HRNR | 0.000% | 0.005% | 0.000% | 0.009% |
| IGHA1 | 1.570% | 0.950% | 0.530% | 0.710% |
| IGHA2 | 0.020% | 0.010% | 0.000% | 0.010% |
| IGHG1 | 3.610% | 1.550% | 1.200% | 0.980% |
| IGHG2 | 1.030% | 0.040% | 0.040% | 0.090% |
| IGHG3 | 3.260% | 0.300% | 0.220% | 0.330% |
| IGHG4 | 0.930% | 0.010% | 0.030% | 0.020% |
| IGHM | 0.700% | 0.790% | 0.400% | 0.330% |
| IGHV1-3; IGHV1-69-2 | 0.020% | 0.000% | 0.000% | 0.000% |
| IGHV3-15; IGHV3-73 | 0.240% | 0.000% | 0.000% | 0.000% |
| IGHV3-21 | 0.400% | 0.010% | 0.010% | 0.010% |
| IGHV3-23 | 0.010% | 0.000% | 0.000% | 0.000% |
| IGHV3-35 | 0.010% | 0.000% | 0.000% | 0.000% |
| IGHV3-38 | 0.020% | 0.000% | 0.000% | 0.000% |
| IGHV3-49 | 0.010% | 0.000% | 0.000% | 0.000% |
| IGHV3-64D | 0.010% | 0.000% | 0.000% | 0.000% |
| IGHV3-66 | 0.240% | 0.000% | 0.000% | 0.000% |
| IGHV3-74; IGHV3-66 | 0.000% | 0.010% | 0.000% | 0.000% |
| IGHV3-9 | 0.000% | 0.000% | 0.000% | 0.000% |
| IGHV4-61; IGHV4-4; IGHV4-31 | 0.000% | 0.000% | 0.000% | 0.000% |
| IGHV5-10-1 | 0.030% | 0.000% | 0.000% | 0.000% |
| IGJ | 0.060% | 0.020% | 0.010% | 0.020% |
| IGKC | 3.740% | 0.790% | 0.460% | 0.390% |
| IGKV A18; IGKV2D-29; IGKV2D-26 | 0.130% | 0.000% | 0.000% | 0.000% |
| IGKV1-5 | 0.050% | 0.000% | 0.000% | 0.000% |
| IGKV2-24; IGKV2D-24 | 0.010% | 0.000% | 0.000% | 0.000% |
| IGKV2-30 | 0.020% | 0.000% | 0.000% | 0.000% |
| IGKV2D-28; IGKV2-40 | 0.080% | 0.010% | 0.010% | 0.010% |
| IGKV3-20 | 0.220% | 0.000% | 0.000% | 0.000% |
| IGKV3D-11 | 0.250% | 0.040% | 0.040% | 0.070% |
| IGKV3D-15 | 0.020% | 0.000% | 0.000% | 0.000% |
| IGKV3D-15; IGKV3D-7 | 0.000% | 0.020% | 0.000% | 0.000% |
| IGKV3D-20 | 0.010% | 0.000% | 0.000% | 0.000% |
| IGKV3D-7 | 0.190% | 0.000% | 0.000% | 0.000% |
| IGKV4-1 | 0.150% | 0.000% | 0.000% | 0.010% |
| IGLC3 | 3.500% | 0.000% | 0.000% | 0.000% |
| IGLC6 | 0.000% | 1.070% | 0.720% | 0.860% |
| IGLC7 | 0.000% | 0.000% | 0.000% | 0.000% |
| IGLC7 | 0.010% | 0.000% | 0.000% | 0.000% |
| IGLL5; IGLC1 | 0.020% | 0.480% | 0.240% | 0.340% |
| IGLV1-44 | 0.050% | 0.000% | 0.000% | 0.000% |
| IGLV1-51 | 0.040% | 0.000% | 0.000% | 0.000% |
| IGLV2-11 | 0.030% | 0.000% | 0.000% | 0.000% |
| IGLV2-14 | 0.020% | 0.000% | 0.000% | 0.000% |
| IGLV3-10 | 0.000% | 0.010% | 0.010% | 0.000% |
| IGLV3-10 | 0.060% | 0.000% | 0.000% | 0.000% |
| IGLV3-16 | 0.030% | 0.000% | 0.000% | 0.000% |
| IGLV3-19 | 0.030% | 0.000% | 0.000% | 0.000% |
| IGLV3-9 | 0.010% | 0.000% | 0.000% | 0.000% |
| IGLV4-69 | 0.000% | 0.000% | 0.000% | 0.010% |
| IGLV5-45 | 0.010% | 0.000% | 0.000% | 0.000% |
| IGLV7-46 | 0.070% | 0.000% | 0.000% | 0.010% |
| ITIH1 | 0.040% | 0.180% | 0.270% | 1.100% |
| ITIH2 | 0.100% | 0.560% | 0.840% | 3.020% |
| ITIH3 | 0.000% | 0.010% | 0.010% | 0.590% |
| ITIH4 | 0.080% | 0.380% | 0.070% | 0.160% |
| KLKB1 | 0.019% | 0.002% | 0.000% | 0.001% |
| KNG1 | 0.150% | 0.657% | 0.509% | 0.445% |
| KRT1 | 0.000% | 1.170% | 1.273% | 1.540% |
| KRT10 | 0.000% | 1.026% | 0.918% | 0.873% |
| KRT14; KRT16; KRT17 | 0.000% | 0.003% | 0.002% | 0.028% |
| KRT2 | 0.000% | 0.155% | 0.185% | 0.263% |
| KRT5 | 0.000% | 0.012% | 0.015% | 0.047% |
| KRT9 | 0.000% | 0.195% | 0.222% | 0.280% |
| LBP | 0.000% | 0.020% | 0.000% | 0.000% |
| LCAT | 0.000% | 0.000% | 0.000% | 0.000% |
| LGALS3BP | 0.000% | 0.000% | 0.000% | 0.000% |
| LPA | 0.000% | 0.260% | 0.230% | 0.250% |
| LRG1 | 0.020% | 0.000% | 0.000% | 0.000% |
| LUM | 0.010% | 0.010% | 0.020% | 0.080% |
| MYO5A | 0.000% | 0.180% | 0.386% | 0.265% |
| OPTN | 0.000% | 0.000% | 0.020% | 0.000% |
| ORM1 | 1.000% | 1.230% | 0.590% | 0.430% |
| ORM2 | 0.200% | 0.110% | 0.030% | 0.020% |
| PCSK9 | 0.000% | 0.000% | 0.000% | 0.010% |
| PDZK1P1; PDZK1 | 0.010% | 0.000% | 0.000% | 0.000% |
| PF4; PF4V1 | 0.220% | 0.000% | 0.000% | 0.000% |
| PGLYRP2 | 0.000% | 0.010% | 0.010% | 0.020% |
| PGLYRP2 | 0.010% | 0.000% | 0.000% | 0.000% |
| PLG | 0.155% | 0.251% | 0.378% | 0.270% |
| PON1 | 0.000% | 0.070% | 0.070% | 0.350% |
| PON1 | 0.050% | 0.000% | 0.000% | 0.000% |
| PROC | 0.000% | 0.000% | 0.000% | 0.080% |
| PROS1 | 0.000% | 0.020% | 0.010% | 0.360% |
| PROS1 | 0.005% | 0.019% | 0.011% | 0.365% |
| PROZ | 0.000% | 0.000% | 0.000% | 0.140% |
| RBP4 | 0.070% | 0.090% | 0.050% | 0.090% |
| SAA1; SAA2 | 0.070% | 0.030% | 0.050% | 0.000% |
| SAA4 | 0.070% | 0.110% | 0.180% | 0.020% |
| SEPP1 | 0.000% | 0.000% | 0.000% | 0.010% |
| SERPINA1 | 2.240% | 3.680% | 1.050% | 1.950% |
| SERPINA10 | 0.000% | 0.000% | 0.000% | 0.060% |
| SERPINA3 | 0.190% | 0.620% | 0.850% | 0.240% |
| SERPINA6 | 0.000% | 0.010% | 0.000% | 0.000% |
| SERPINC1 | 0.000% | 0.450% | 0.190% | 0.180% |
| SERPIND1 | 0.040% | 0.000% | 0.000% | 0.000% |
| SERPIND1; SERPINC1 | 0.200% | 0.183% | 0.288% | 0.178% |
| SERPINF1 | 0.010% | 0.000% | 0.000% | 0.000% |
| SERPINF1; SERPINF2 | 0.020% | 0.076% | 0.153% | 0.035% |
| SERPINF2 | 0.000% | 0.020% | 0.080% | 0.080% |
| SERPING1 | 0.020% | 0.200% | 0.180% | 0.100% |
| SPP2 | 0.000% | 0.000% | 0.050% | 0.200% |
| SPTLC3; SPTLC2 | 0.000% | 0.280% | 0.250% | 0.240% |
| SUSD2 | 0.000% | 0.000% | 0.010% | 0.000% |
| TF | 3.310% | 0.470% | 0.530% | 0.430% |
| THBS1 | 0.000% | 0.000% | 0.000% | 0.050% |
| THBS4 | 0.000% | 0.000% | 0.000% | 0.050% |
| TMEM198 | 0.010% | 0.000% | 0.000% | 0.000% |
| TTR | 0.190% | 0.040% | 0.090% | 0.030% |
| Vitronectin | 0.310% | 1.300% | 13.000% | 15.700% |
| ZNF554 | 0.000% | 0.000% | 0.000% | 0.010% |

Transcriptomic Screening and Selection Matrix for siRNA Therapeutic Targets

**Data Availability and Source Identification.** The bioinformatic analyses presented in this study are based on pre-existing transcriptomic datasets retrieved from the Gene Expression Omnibus (GEO) database. These public resources were utilized for integrative re-analysis. The accession numbers and study details are as follows: Dataset 1: GSE76309 (Whole liver RNA-seq, ARE-Del-/- mice). Dataset 2: GSE226946 (Bulk RNA-seq, Kupffer cells from CCl_4_-induced fibrotic livers). All data processing and integration were performed following standard bioinformatic pipelines as detailed in the subsequent sections.

**Integrative Transcriptomic Analysis and Pathway Enrichment.** To identify liver fibrosis drivers and macrophage-specific dysfunction, we performed an integrative analysis of two transcriptomic datasets: GSE76309 (whole liver RNA-seq from ARE-Del-/-mice) and GSE226946 (bulk RNA-seq of KCs from CCl_4_-induced fibrotic livers). Differential gene expression analysis was conducted in RStudio. We first mapped the fibrotic liver transcriptome by identifying genes significantly down-regulated in both datasets (adjusted p-value < 0.05, log2FC > 1).

Pathway enrichment was performed using Metascape (Metascape provides a biologist-oriented resource for the analysis of systems-level datasets. Y. Zhou, B. Zhou, L. Pache, M. Chang, A. H. Khodabakhshi, O. Tanaseichuk, et al. Nature communications 2019 Vol. 10 Issue 1 Pages 1523). Terms meeting the criteria of p-value < 0.01, minimum count of 3, and enrichment factor > 1.5 were aggregated into clusters based on gene membership similarity using k-scores. Statistical significance was calculated using the cumulative hypergeometric distribution, with a Benjamini-Hochberg adjustment. To isolate core pathological drivers, we prioritized clusters showing statistically significant enrichment across all conditions (ARE-Del-/- male/female, KC-CCl_4_). The top 20 enriched functional pathways are compiled in **Table S2.** The InTerm InList column denotes the number of input genes participating in each pathway, while "%" represents the percentage of provided genes identified within the given ontology term. In this ranking, specific terms marked with an asterisk (*) identify the robust core pathways concurrently shared across all three analyzed conditions: ARE-Del-/- males, ARE-Del-/- females, and CCl_4_-induced KCs. Notably, the "M5885: NABA MATRISOME ASSOCIATED*" cluster emerged as one of these highly conserved, cross-model pathological hubs ($\text{LogP} = -18.10$), highlighting a coordinated activation of extracellular matrix (ECM) components regardless of sex or specific fibrotic stimulus.

**Candidate Prioritization Strategy.** To identify the optimal therapeutic target for siRNA-mediated silencing, we implemented a multi-stage prioritization pipeline:

Cross-Dataset Integration: We intersected genes consistently up-regulated (p-value < 0.15, log2FC > 1) in the ARE-Del model (merged male and female datasets) with those down-regulated in fibrotic KCs (adjusted p-value < 0.05, log2FC > 1). This filter ensured candidates were pathologically relevant to both the global liver microenvironment and the macrophage-specific compartment, specifically aligning with the ECM and cell-substrate interaction pathways identified in the previous section.

Candidate Ranking: The identified core genes were screened and ranked using a comprehensive mathematical metric designated as the **Balanced Relevance Score** (as detailed in **Table S3**. This metric was calculated as follows:

Balanced Relevance Score = n GO terms $\times$ Max Bonus $\times$ Transcriptional Coherence

The formula integrates two primary biological dimensions:

-Functional Hubness: Genes were ranked by their connectivity within the Gene Ontology network (n GO terms).

-Biological Relevance: A categorical weighted bonus multiplier was applied to genes linked to specific pathological drivers (Bonus Keys: 2.0× for ECM & Remodeling; 2.0× for Inflammation; 1.5× for Immune Activation; 1.0× for Transport & Regulation).

Selection Criteria: We applied a 'Maximum Relevance' filter, prioritizing genes with a top-tier biological relevance score (3.0x) and involved in multiple clusters (ECM/Matrisome and Inflammation). Preference was given to genes displaying consistent up-regulation across all three analyzed models (ARE-Del-/- male/female, KC-CCl₄), with specific emphasis on the female cohort, which exhibits a more pronounced fibrotic phenotype.

**Table S2.** Pathway and process enrichment analysis of significantly downregulated genes.

**Table S3.** Screening matrix and prioritization ranking of the top candidate hub genes for siRNA-mediated therapeutic targeting.

Gene Expression and Correlation Analysis by qPCR

To assess the transcriptional effects of TIMP1 silencing and identify genes co-regulated with Timp1, we performed a targeted gene expression analysis using qPCR on total liver RNA extracted from WT and ARE-Del-/- mice, samples were obtained from both sexes, across different treatment conditions (PBS, scr-LNPS, and siTIMP1-LNPs), and processed individually to preserve biological replicates. Total RNA was isolated using TRIzol™ reagent (Invitrogen), and 1μg of RNA per sample was reverse-transcribed using the High-Capacity cDNA Reverse Transcription Kit (Applied Biosystems), following the manufacturer’s instructions. qPCR was performed using a QuantStudio 5 Real-Time PCR System (Applied Biosystems). Two strategies were employed:

**TaqMan™ Array Cards** (Custom 96-well Format): A customized TaqMan array was designed (Applied Biosystems) to include genes related to fibrosis, extracellular matrix remodeling, immune signaling, apoptosis, and inflammation, as well as four endogenous controls (*Hprt*, *Gapdh*, *18S rRNA*, and *Gusb*; full list in **Table S4**). Amplification was performed using TaqMan™ Fast Advanced Master Mix and the QuantStudio™ 5 Real-Time PCR System. Each sample was run on an individual port of the array card. Data were analyzed using the comparative Ct method (ΔΔCt), with Hprt selected as the primary reference gene after assessment of housekeeping stability.

**SYBR™ Green Assays**: Additional targets not included in the array were analyzed using PowerUp™ SYBR™ Green Master Mix (Applied Biosystems). Primer sequences were validated for specificity and efficiency. Reactions were performed in technical duplicates, and melt curve analysis was conducted to confirm single amplicon generation.

For correlation analysis, ΔCt values were computed and used to generate Pearson correlation matrices between Timp1 and all other genes. Hierarchical clustering and heatmap visualization were performed in R (v4.2.0) using the pheatmap package. Genes were grouped by correlation patterns, and clusters reflecting positively or negatively associated genes were identified. This analysis allowed identification of functionally co-regulated gene sets potentially involved in TIMP1–mediated pathways.

**Table S4.** Custom Taqman Array Cards genes list

| Gene Symbol | Probe Id | Gene Name |
| --- | --- | --- |
| 18s Rrna | Hs99999901_s1 | 18s ribosomal RNA |
| Gapdh | Mm99999915_g1 | Glyceraldehyde-3-phosphate dehydrogenase |
| Hprt | Mm00446968_m1 | Hypoxanthine guanine phosphoribosyl transferase |
| Gusb | Mm00446953_m1 | Beta-glucuronidase |
| Acta2 | Mm00725412_s1 | Actin alpha 2, smooth muscle |
| Arg1 | Mm00475988_m1 | Arginase 1 |
| B2m | Mm00437762_m1 | Beta-2 microglobulin |
| Bax | Mm00432050_m1 | Bcl2 associated X protein |
| Bcl2 | Mm00477631_m1 | B-cell leukemia/lymphoma 2 |
| Bid | Mm00432073_m1 | BH3 interacting domain death agonist |
| Casp1 | Mm00438023_m1 | Caspase 1 |
| Casp3 | Mm01195085_m1 | Caspase 3 |
| Casp8 | Mm00802247_m1 | Caspase 8 |
| Casp9 | Mm00516563_m1 | Caspase 9 |
| Ccl2 | Mm00441242_m1 | C-C motif chemokine ligand 2 |
| Ccl3 | Mm00441258_m1 | C-C motif chemokine ligand 3 |
| Ccl5 | Mm01302428_m1 | C-C motif chemokine ligand 5 |
| Ccr2 | Mm99999051_gH | C-C motif chemokine receptor 2 |
| Ccr4 | Mm00438271_m1 | C-C motif chemokine receptor 4 |
| Cdkn1b | Mm00438168_m1 | Cyclin-dependent kinase inhibitor 1B (p27 Kip1) |
| Col1a2 | Mm00483888_m1 | Collagen type I alpha 2 chain |
| Col3a1 | Mm01254476_m1 | Collagen type III alpha 1 chain |
| Cxcl10 | Mm00445235_m1 | C-X-C motif chemokine ligand 10 |
| Cxcl9 | Mm00434946_m1 | C-X-C motif chemokine ligand 9 |
| Cxcr4 | Mm01292123_m1 | C-X-C motif chemokine receptor 4 |
| Fas | Mm00433237_m1 | Fas cell surface death receptor |
| Fasl | Mm00438864_m1 | Fas ligand |
| Fn1 | Mm01256744_m1 | Fibronectin 1 |
| Fn1 | Mm01256734_m1 | Fibronectin 1 |
| Foxp3 | Mm00475162_m1 | Forkhead box P3 |
| Ifnb1 | Mm00439552_s1 | Interferon beta 1 |
| Ifne | Mm00616542_s1 | Interferon epsilon |
| Ifng | Mm00801778_m1 | Interferon gamma |
| Ifnz | Mm02525738_g1 | Interferon zeta |
| Il10 | Mm00439616_m1 | Interleukin 10 |
| Il1a | Mm00439620_m1 | Interleukin 1 alpha |
| Il1b | Mm00434228_m1 | Interleukin 1 beta |
| Il1r1 | Mm00434237_m1 | Interleukin 1 receptor type I |
| Il1rap | Mm00492638_m1 | Interleukin 1 receptor accessory protein |
| Il2 | Mm00434256_m1 | Interleukin 2 |
| Il3 | Mm00439631_m1 | Interleukin 3 |
| Il6 | Mm00446190_m1 | Interleukin 6 |
| Ipo8 | Mm01255158_m1 | Importin 8 |
| Irf1 | Mm01288580_m1 | Interferon regulatory factor 1 |
| Irf2 | Mm00515206_m1 | Interferon regulatory factor 2 |
| Irf3 | Mm00516784_m1 | Interferon regulatory factor 3 |
| Irf5 | Mm00496477_m1 | Interferon regulatory factor 5 |
| Irf7 | Mm00516793_g1 | Interferon regulatory factor 7 |
| Irf9 | Mm00492679_m1 | Interferon regulatory factor 9 |
| Jak1 | Mm00600614_m1 | Janus kinase 1 |
| Jak2 | Mm01208489_m1 | Janus kinase 2 |
| Jak3 | Mm00439973_m1 | Janus kinase 3 |
| Jun | Mm00495062_s1 | Jun proto-oncogene |
| Mapk1 | Mm00442479_m1 | Mitogen-activated protein kinase 1 |
| Mapk8 | Mm00489514_m1 | Mitogen-activated protein kinase 8 (JNK1) |
| Mmp12 | Mm00500554_m1 | Matrix metallopeptidase 12 |
| Mmp3 | Mm00440295_m1 | Matrix metallopeptidase 3 |
| Mmp9 | Mm00442991_m1 | Matrix metallopeptidase 9 |
| Myc | Mm00487804_m1 | MYC proto-oncogene |
| Myd88 | Mm00440338_m1 | Myeloid differentiation primary response 88 |
| Nfkb1 | Mm00476361_m1 | Nuclear factor kappa B subunit 1 |
| Nod1 | Mm00805062_m1 | Nucleotide-binding oligomerization domain 1 |
| Nod2 | Mm00467543_m1 | Nucleotide-binding oligomerization domain 2 |
| Nos2 | Mm00440485_m1 | Nitric oxide synthase 2, inducible |
| Pdgfa | Mm01205760_m1 | Platelet-derived growth factor alpha |
| Serpine1 | Mm00435860_m1 | Serpin family E member 1 (PAI-1) |
| Serpinh1 | Mm00438058_g1 | Serpin family H member 1 (HSP47) |
| Smad2 | Mm00487530_m1 | SMAD family member 2 |
| Smad3 | Mm00489637_m1 | SMAD family member 3 |
| Smad3 | Mm01170760_m1 | SMAD family member 3 |
| Smad4 | Mm03023996_m1 | SMAD family member 4 |
| Smad7 | Mm00484741_m1 | SMAD family member 7 |
| Socs1 | Mm00782550_s1 | Suppressor of cytokine signaling 1 |
| Socs2 | Mm00850544_g1 | Suppressor of cytokine signaling 2 |
| Stat1 | Mm00439518_m1 | Signal transducer and activator of transcription 1 |
| Stat3 | Mm00456961_m1 | Signal transducer and activator of transcription 3 |
| Stat4 | Mm00448890_m1 | Signal transducer and activator of transcription 4 |
| Stat6 | Mm01160477_m1 | Signal transducer and activator of transcription 6 |
| Tgfb1 | Mm01178820_m1 | Transforming growth factor beta 1 |
| Tgfb2 | Mm00436955_m1 | Transforming growth factor beta 2 |
| Timp1 | Mm01341361_m1 | Tissue inhibitor of metalloproteinases 1 |
| Tirap | Mm00446502_m1 | Toll-interleukin 1 receptor domain-containing |
| Tlr1 | Mm00446095_m1 | Toll-like receptor 1 |
| Tlr2 | Mm00442346_m1 | Toll-like receptor 2 |
| Tlr3 | Mm00628112_m1 | Toll-like receptor 3 |
| Tlr4 | Mm00445273_m1 | Toll-like receptor 4 |
| Tlr5 | Mm00546288_s1 | Toll-like receptor 5 |
| Tlr6 | Mm02529782_s1 | Toll-like receptor 6 |
| Tlr8 | Mm04209873_m1 | Toll-like receptor 8 |
| Tlr9 | Mm00446193_m1 | Toll-like receptor 9 |
| Tnfrsf11a | Mm00437132_m1 | TNF receptor superfamily member 11a (RANK) |
| Tnfrsf1a | Mm00441883_g1 | TNF receptor superfamily member 1a |
| Tnfsf10 | Mm01283606_m1 | TNF-related apoptosis-inducing ligand (TRAIL) |
| Tollip | Mm00445841_m1 | Toll interacting protein |
| Vcam1 | Mm00449197_m1 | Vascular cell adhesion molecule 1 |
| Vegfa | Mm01281449_m1 | Vascular endothelial growth factor A |

**Table S5.** Primer sequences used for SYBR™ Green qPCR assays

| Gene | Forward Primer (5′→3′) | Reverse Primer (5′→3′) |
| --- | --- | --- |
| Acta2 | GCACCCAGCATGAAGATCAAG | TCTGCTGGAAGGTAGACAGCGAAG |
| Arg1 | CATGGGCAACCTGTGTCCTT | TCCTGGTACATCTGGGAACTTTC |
| CD206 | CATGAGGCTTCTCCTGCTTC | CAGCGCTTGTGATCTTCATT |
| Col1a1 | ACCTGTGTGTTCCCTACTCA | GACTGTTGCCTTCGCCTCTG |
| Col1a2 | TGAAGTGGGTCTTCCAGGTCT | CACCCTTGTTACCGGATTCTCCTT |
| Col3a1 | GCCCACAGCCTTCTACAC | CCAGGGTCACCATTTCTC |
| Col4a1 | ATCCTATTGAACCCGGAGAGC | TGACATGCGACTCAAAGGTG |
| Cxcl9 | TGGGAAAAGAAGATCAGCCAA | CCTGGGATTTGGTGACGTGA |
| Cxcl10 | TGTTGAGATCATTGCCACGATG | AGATGGTGGTTAAGTTCGTCCT |
| Hprt | GTTCTTGCTGACCTGCTGG | CATTTTGGGGCTGTACTGCT |
| IL-6 | TCTCTGGGAAATCGTGGAAA | TCTGCAAGTGCATCATCGTT |
| IL-10 | CGGCTGAGGCGCTGT | TGCCTTGCTCTTATTTTCACAGG |
| iNOS | GACGAGACGGATAGGCAGAG | GTGGGGTTGTTGCTGAACTT |
| Mmp12 | TTGACCCACTTCGCCAAAAG | TCCTGCCTCACATCATACCTC |
| Mmp14 | AGGAGACAGAGGTGATCATCATTG | GTCCCATGGCGTCTGAAGA |
| Mmp15 | ATCCCCTATGACCGCATTGAC | CCCCTGCCAGACACTGATG |
| Mmp3 | TAAAGACAGGCACTTTTGGC | GTAACCTCATATGCAGCATCC |
| Mmp9 | ACCTGAAAACCTCCAACCTC | TCGAATGGCCTTTAGTGTCTG |
| Timp1 | CTCAAAGACCTATAGTGCTGGC | CAAAGTGACGGCTCTGGTAG |
| Timp2 | GTCAGCCTTCTTACGGGTCC | GGGCTGTGAGTGCAAGATCA |
| Timp3 | GGCCTCAATTACCGCTACCA | CTGATAGCCAGGGTACCCAAAA |
| Timp4 | TGCAGAGGGAGAGCCTGAA | GGTACATGGCACTGCATAGCA |

**Table S6.** *Welfare Assessment Table*

To monitor animal well-being throughout the study, both ARE-Del-/- and wild-type (WT) mice were evaluated three times per week over the 5-week experimental window covering the entire 12-injection treatment period for the treated groups. The table reports aggregate welfare scores across all experimental groups: PBS, scr-LNPs, siTIMP1-LNPs, and untreated WT controls. Assessed parameters included body weight, hydration status, coat condition, nasal/ocular secretions, posture, and social interaction. Respiratory function was not evaluated. One female ARE-Del⁻/⁻ mouse in the PBS group died during the study, showing signs of progressive disease, including weight loss and reduced interaction. All other animals—including those receiving chronic siTIMP1-LNP treatment—maintained stable clinical conditions throughout the experimental window.

| Clinical Parameter | Partial Score | Observed Distribution (%) |
| --- | --- | --- |
| Body weight | 0 = No loss | 94% |
|  | 1 = 5–10% loss | 3% |
|  | 2 = >10% loss | 3% *(1 female – deceased)* |
| Hydration | 0 = Normal | 100% |
|  | 1 = Moderate loss | 0% |
|  | 2 = Severe loss | 0% |
| Coat condition | 0 = Smooth | 96% |
|  | 1 = Ruffled | 4% |
|  | 2 = Matted | 0% |
| Nasal/ocular secretions | 0 = Absent | 100% |
|  | 1 = Mild | 0% |
|  | 2 = Severe | 0% |
| Gait/posture | 0 = Normal | 100% |
|  | 1 = Slightly altered | 0% |
|  | 2 = Abnormal | 0% |
| Social interaction | 0 = Active | 97% |
|  | 1 = Isolated | 3% *(observed in the deceased female before death)* |
|  | 2 = Apathic | 0% |
